# Gene Expression Analysis of the Pre-Diabetic Pancreas to Identify Pathogenic Mechanisms and Biomarkers of Type 1 Diabetes

**DOI:** 10.3389/fendo.2020.609271

**Published:** 2020-12-23

**Authors:** Linda Yip, Rebecca Fuhlbrigge, Reem Alkhataybeh, C. Garrison Fathman

**Affiliations:** Division of Immunology and Rheumatology, Department of Medicine, Stanford University, Stanford, CA, United States

**Keywords:** gene expression, type 1 diabetes, auto-antibody positive, non-obese diabetic mice, pancreas, biomarker, FCGR2B

## Abstract

Type 1 Diabetes (T1D) occurs as a result of the autoimmune destruction of pancreatic β-cells by self-reactive T cells. The etiology of this disease is complex and difficult to study due to a lack of disease-relevant tissues from pre-diabetic individuals. In this study, we performed gene expression analysis on human pancreas tissues obtained from the Network of Pancreatic Organ Donors with Diabetes (nPOD), and showed that 155 genes were differentially expressed by ≥2-fold in the pancreata of autoantibody-positive (AA+) at-risk individuals compared to healthy controls. Only 48 of these genes remained changed by ≥2-fold in the pancreata of established T1D patients. Pathway analysis of these genes showed a significant association with various immune pathways. We were able to validate the differential expression of eight disease-relevant genes by QPCR analysis: A significant upregulation of *CADM2*, and downregulation of *TRPM5, CRH, PDK4, ANGPL4, CLEC4D, RSG16*, and *FCGR2B* was confirmed in the pancreata of AA+ individuals versus controls. Studies have already implicated *FCGR2B* in the pathogenesis of disease in non-obese diabetic (NOD) mice. Here we showed that *CADM2, TRPM5, PDK4*, and *ANGPL4* were similarly changed in the pancreata of pre-diabetic 12-week-old NOD mice compared to NOD.B10 controls, suggesting a possible role for these genes in the pathogenesis of both T1D and NOD disease. The loss of the leukocyte-specific gene, *FCGR2B*, in the pancreata of AA+ individuals, is particularly interesting, as it may serve as a potential whole blood biomarker of disease progression. To test this, we quantified *FCGR2B* expression in peripheral blood samples of T1D patients, and AA+ and AA- first-degree relatives of T1D patients enrolled in the TrialNet Pathway to Prevention study. We showed that *FCGR2B* was significantly reduced in the peripheral blood of AA+ individuals compared to AA- controls. Together, these findings demonstrate that gene expression analysis of pancreatic tissue and peripheral blood samples can be used to identify disease-relevant genes and pathways and potential biomarkers of disease progression in T1D.

## Introduction

Type 1 Diabetes (T1D) results from the autoimmune and chronic destruction of pancreatic β-cells by self-reactive T cells. It is the most common autoimmune disease in children, affecting approximately 1 in 500 in the United States, and the incidence is growing each year (1). The etiology of this disease is complex, and involves a strong genetic component and a number of possible environmental triggers. Genome-wide association studies (GWAS) have identified >60 polymorphisms that contribute to the genetic susceptibility of T1D (2, 3) and various environmental triggers have been shown to play a role in disease onset (4–6). The pathogenesis of this disease, however, remains unclear due to the difficulty in identifying pre-diabetic individuals and in obtaining biological specimens from such individuals.

Animal models of T1D have been crucial in understanding the mechanisms involved in the pathogenesis of T1D, but there are notable differences in human disease development. In humans, β-cell destruction occurs months to years before the onset of hyperglycemia. Individuals are asymptomatic, and develop autoantibodies (AAs) against various islet antigens including glutamic acid decarboxylase (GAD), zinc transporter 8 (ZnT8), insulin, and islet antigen 2 (IA-2) during the pre-hyperglycemic stage of disease. The presence and number of AAs detected in the serum is currently the most reliable biomarker for assessing the disease risk. However, there is a need for additional early, robust and reproducible biomarkers of disease progression.

The pancreata of at-risk AA+ individuals are significantly smaller than those of healthy individuals (7, 8), and analysis of pancreas sections demonstrate increased CD4+ and CD11c+ immune cell infiltration in the exocrine pancreas of these individuals (9). The islets of at-risk individuals differ functionally and morphologically from healthy controls (10), but insulitis is only detected in a small percentage of AA+ individuals (11, 12). In contrast, the islets of non-obese diabetic (NOD) mice, a well-established mouse model of T1D, develop large peri-insulitic lesions beginning at 4 weeks of age. Destructive insulitis occurs at ~12 weeks of age, and hyperglycemia follows at ~16 to 25 weeks of age (13).

We and others have shown that the pancreata and islets of rodent models of T1D differ from those of healthy controls, and that these differences are reflected in the gene expression profile during the progression of disease (13–15). Gene expression data support the upregulation of glucagon and insulin secretion, β-cell hyperactivation and/or ER stress playing a role during different stages of disease development (13–15). Currently, it is unclear if similar changes in gene expression are observed in the pancreata of pre-diabetic humans after seroconversion preceding hyperglycemia.

Human pancreatic tissues from healthy controls, AA+ and T1D patients are available through the Juvenile Diabetes Research Foundation Network for Pancreatic Organ Donors with Diabetes (JDRF nPOD; www.jdrfnpod.org). These samples are collected from deceased donors and processed under well-defined standard operating procedures. However, due to the high level of pancreatic ribonucleases and tissue recovery time, RNA degradation occurs (16–18). Nevertheless, studies have shown that the impact of RNA degradation on the assessment of gene expression by microarray analysis is minimal, and largely offset by biological differences between samples (19).

In this study, we obtained pancreas samples of at-risk AA+ individuals, T1D patients, and healthy controls from nPOD and performed gene expression analyses. We then compared the genes that were differentially expressed in pre-diabetic AA+ individuals with those that were differentially expressed in the pancreata of NOD mice. The overlapping genes may be pathogenic and play a role in the development of T1D and NOD disease. We identified a number of biologically relevant genes that were changed in the pancreata of AA+ individuals. One gene, in particular, is expressed in leukocytes and could serve as a potential biomarker of disease progression that may be monitored in blood samples of individuals with a genetic risk for developing T1D. This gene, *FCGR2B*, encodes the CD32B receptor, an inhibitory low affinity receptor for the Fc region of IgG complexes (20, 21). Changes to *FCGR2B*/CD32B expression have been associated with multiple autoimmune diseases in humans (22–25), and have been suggested to underlie the autoimmune susceptibility of several strains of mice, including NOD mice (26, 27).

In this study, we also examined the potential of *FCGR2B* as a candidate biomarker of disease progression, by comparing *FCGR2B* expression in peripheral blood RNA samples of T1D patients, AA+ individuals who later developed T1D (AA+ progressors), and AA- first-degree relatives of T1D patients (AA- FDRs) obtained from the National Institute of Health TrialNet repository.

## Materials and Methods

### Human Samples

#### Pancreas

Human pancreas samples were obtained through the JDRF network for Pancreatic Organ Donors with Diabetes (nPOD). Patient information for the final samples used for this study is shown on **Tables 1A** and **B**, and standard operating procedures for tissue processing are available at https://www.jdrfnpod.org/for-investigators/standard-operating-procedures/. Tissues were obtained from cadaveric donors that died of various causes, and AA+ individuals were identified by autoantibody screening after death. Thus, the length of islet autoimmunity in AA+ individuals is unknown. The pancreatic tissues used for this study were minced, placed in cryovials containing RNAlater, incubated at room temperature for 15 min and then snap frozen and stored at -80°C until shipment. RNA was extracted as described below.

**Table 1A:** Patient information for nPOD pancreata samples.

nPOD ID	Serum AA*	Region	Age^†^	Sex	BMI	Ethnicity	Cause of Death^††^	C-peptide (ng/ml)	Pancreas Weight (g)	RNA RIN
**Normal Controls**										
6102	Negative	body	45	F	35.1	Caucasian	CVS	0.55	87.32	4.6
6172	Negative	tail	19	F	32.4	Caucasian	CVS	8.02	59.7	5.8
6179	Negative	tail	21	F	20.7	Caucasian	HT	2.74	72.4	5.2
6227	Negative	body	17	F	26.4	Caucasian	CVS	2.75	60.4	5.6
6229	Negative	body	31	F	26.9	Caucasian	HT	6.23	45.6	7
6234	Negative	body	20	F	25.6	Caucasian	HT	6.89	49.86	3.7
6253	Negative	body	19	F	34.3	African American	HT	7.22	102.31	5.3
**AA+ subjects**										
6027	ZnT8A+	tail	18	M	19.9	Caucasian	n/a	n/a	52.9	4.4
6090	GADA+	head	2	M	18.8	Hispanic/Latino	HT	5.34	19.3	5.5
6123	GADA+	head	23	F	17.6	Caucasian	HT	2.01	48.34	7.6
6170	GADA+	head	34	F	36.9	African American	A	4.29	110.3	7.6
6184	GADA+	body	47	F	27	Hispanic/Latino	HT	3.42	71.8	4.4
6197	GADA+ IA-2A+	tail	22	M	28.2	African American	HT	17.48	73.3	8.1
**Established T1D subjects**										
6088	GADA+ IA-2A+ ZnT8A+ mIAA+	head	31 (T1D: 5)	M	27	Caucasian	HT	<0.05	32	5.5
6143	IA-2A+ mIAA+	head	32 (T1D: 7)	F	26.1	Caucasian	A	<0.05	35.7	8.5
6148	GADA+ mIAA+	head	17 (T1D: 7)	M	23.9	Caucasian	A	0.06	26.36	5.0
6161	IA-2A+ mIAA+	head	19 (T1D: 7)	F	36.1	Caucasian	CVS	<0.05	39.8	5.0
6180	GADA+ IA-2A+ ZnT8A+ mIAA+	body	27 (T1D: 11)	M	25.9	Caucasian	HT	<0.05	36.7	4.0
6241	mIAA+	body	33 (T1D: 31)	M	18.4	Caucasian	CVS	<0.05	24.92	8.0
6245	GADA+ IA-2A+	body	22 (T1D: 7)	M	23.2	Caucasian	HT	<0.05	31.58	7.2
6258	mIAA+	body	39 (T1D: 37)	F	28.7	Caucasian	HT	<0.05	32.56	5.4
6263	Negative	body	34 (T1D: 21)	M	23.5	Hispanic/Latino	CVS	3.18	51.59	7.8
6266	GADA+ IA-2A+ mIAA+ ZnT8A+	body	30 (T1D: 23)	M	27.1	Caucasian	A	<0.05	51.66	6.6

*GAD, Glutamic acid decarboxylase; IA2+, Insulinoma-associated protein 2; mIAA+, micro-Insulin auto-antibody; ZnT8, Zinc transporter 8 as determined by nPOD. Methods used for AA+ detection are available at: http://www.jdrfnpod.org/for-investigators/standard-operating-procedures/.

^†^For T1D samples, years with T1D are indicated.

^††^Cause of Death: HT, Head trauma; CVS, Cerebrovascular/Stroke; A, Anoxia.

**Table 1B:** HLA Information for nPOD pancreata samples.

nPOD ID	HLA Information
	**Normal Controls**
6102	DRB1*03:01, 04:01 DQA1*03:01, 05:01 DQB1*02:01, 03:01 (DR3, DR4)
6172	DRB1*01:01, 13:03 DQA1*01:01, 05:01 DQB1*03:01, 05:01
HLA information not available for 6179, 6227, 6229, 6234 and 6253	
	**AA+ subjects**
6027	DRB1*03:01/15:01 DQA1*01:02/05:01 DQB1*02:01/06:02 (DR3)
6090	DRB1*04:04/15:01 DQA1*01:02/03:01 DQB1*03:02/06:02 (DR4)
6123	DRB1*08:01/11:01 DQA1*04:01/05:01 DQB1*03:01/04:02
6170	DRB1*04:01/13:03 DQA1*02:01/03:01 DQB1*02:02/03:01 (DR4)
6184	DRB1*04:07/04:07 DQA1*03:01/03:01 DQB1*03:02/03:02 (DR4, DR4)
6197	DRB1*03:02/07:01 DQA1*02:01/04:01 DQB1*02:02/04:02(DR3)
	**Established T1D subjects**
6088	DRB1*01:01/03:01 DQA1*01:01/05:01 DQB1*02:01/05:01 (DR3)
6143	DRB1*03:01/04:01 DQA1*03:01/05:01 DQB1*02:01/03:01 (DR3, DR4)
6148	DRB1*03:01/04:01 DQA1*03:01/05:01 DQB1*02:01/03:02 (DR3, DR4)
6161	DRB1*04:01/07:01 DQA1*02:01/03:01 DQB1*02:02/03:02 (DR4)
6180	DRB1*01:01/03:01 DQA1*01:01/05:01 DQB1*02:01/05:01 (DR3)
HLA information not available for 6241, 6245, 6258, 6263, and 6266.	

#### Whole Blood Samples

Whole blood RNA samples were provided by TrialNet. Samples from AA- FDRs (n=15) and AA+ FDRs who later progressed to hyperglycemia (AA+ progressors, n=20) were from individuals enrolled in the TrialNet Pathway to Prevention Study. Samples from T1D patients (n=10) were obtained from individuals enrolled in the Glutamic Acid Decarboxylase (GAD) Vaccine trial. IRB approval was obtained at the institution where samples were collected. All participants provided written informed consent. Before use, all RNA samples were repurified using the Qiagen RNeasy mini kit. Briefly, 100 μl of RNA (<100 μg) was combined with 350 μl of buffer RLT, mixed, added to 250 μl ethanol, and applied to a RNeasy mini column to bind the RNA. The RNA was washed and eluted according to manufacturer’s instructions. Re-extracted RNA concentrations were measured by Nanodrop and RNA quality was determined by Bioanalyzer analysis (Agilent) and listed in **Table 2**.

**Table 2:** Patient information for TrialNet whole blood RNA samples.

AA- First Degree Relatives							
ID	HLA (DRB1:DQA1:DQB1)*		Serum AA^†^	Age	Sex	Years to Onset	RNA RIN
1	HLAa: 1401:0101:0503 HLAb: 1102:0501:0301		Negative	13	F	–	8.1
2	HLAa: 1501:0102:0602 HLAb: 1301:0103:0603		Negative	9	F	–	8.5
3	HLAa: 0701:0201:0202 HLAb: 0401:0301:0302 (DR4)		Negative	15	M	–	8.2
4	HLAa: 1201:0501:0301 HLAb: 1302:0501:0301		Negative	18	M	–	7.8
5	HLAa: 0101:0101:0501 HLAb: 0102:0101:0501		Negative	12	F	–	7.5
6	HLAa: 0401:0301:0302 (DR4) HLAb: 0701:0201:0202		Negative	18	F	–	8.3
7	No data		Negative	24	F	–	5.6
8	HLAa: 0701:0201:0202 HLAb: 0401:0301:0301 (DR4)		Negative	17	M	**-**	7.1
9	HLAa: 0701:0201:0202 HLAb: 0701:0201:0202		Negative	7	F	**-**	8.1
10	HLAa: 0401:0301:0302 (DR4) HLAb: 0301:0501:0201(DR3)		Negative	12	M	**-**	7.8
11	HLAa: 0408:0301:0301 HLAb: 0301:0501:0201 (DR3)		Negative	45	M	–	6.2
12	No data		Negative	34	M	–	7.7
13	HLAa: 1302:0102:0604 HLAb: 0101:0101:0502		Negative	9	M	–	8.4
14	HLAa: 1104:0501:0301 HLAb: 0301:0501:0201 (DR3)		Negative	43	F	–	7.4
15	HLAa: 0401:0301:0301 (DR4) HLAb: 0401:0301:0302 (DR4)		Negative	10	M	–	8.8
			**Average ± SE:**	**19.1 ± 3.1**		**-**	**7.7 ± 0.2**
**AA+ First Degree Relatives that later progressed to T1D**							
**ID**		Serum AA*		**Age**	**Sex**	**Years to Onset**	**RNA RIN**
1	HLAa: 0101:0101:0501 HLAb: 0401:0301:0302 (DR4)	ICA512+		5.5	M	3.0	7.1
2	HLAa: 0301:0501:0201 (DR3) HLAb: 0404:0301:0302 (DR4)	GAD65+		43.6	F	3.9	8.5
3	HLAa: 0301:0501:0201 (DR3) HLAb: 0301:0501:0201 (DR3)	GAD65+		11.8	M	2.1	9.3
4	HLAa: 0401:0301:0302 (DR4) HLAb: 0401:0301:0302 (DR4)	ICA+		23.4	M	1.8	9.2
5	HLAa: 0401:0301:0302 (DR4) HLAb: 0404:0301:0302 (DR4)	GAD65+		40.6	F	3.7	8.8
6	HLAa: 0404:0301:0302 (DR4) HLAb: 0801:0401:0402	GAD65+ GAD65H+		10.0	M	3.3	9.4
7	HLAa: 0301:0501:0201 (DR3) HLAb: 1501:0102:0602	ICA512+		13.5	M	2.6	6.1
8	HLAa: 0301:0501:0201 (DR3) HLAb: 0301:0501:0201 (DR3)	GAD65+ ICA+		41.7	F	3.0	7.0
9	HLAa: 0901:0301:0303 HLAb: 0301:0501:0201 (DR3)	GAD65+ GAD65H+		50.0	M	1.1	7.2
10	No data	GAD65+		4.9	F	2.0	10
11	HLAa: 0401:0301:0302 (DR4) HLAb: 0801:0401:0402	GAD65+ ICA+		8.3	M	3.8	9.8
12	No data	GAD65+		12.6	F	3.4	9.7
13	HLAa: 0401:0301:0302 (DR4) HLAb: 0301:0501:0201(DR3)	GAD65+ ICA+		11.4	M	2.4	9
14	HLAa: 0301:0501:0201 (DR3) HLAb: 0404:0301:0302 (DR4)	GAD65+ ICA+		6.5	M	5.5	8.7
15	HLAa: 0301:0501:0201 (DR3) HLAb: 1302:0102:0604	GAD65+		45.5	M	3.1	8.7
16	HLAa: 0103:0101:0501 HLAb: 0401:0301:0302 (DR4)	ICA512+		23.4	M	3.0	7.5
17	HLAa: 0404:0301:0302 (DR4) HLAb: 1101:0501:0301	GAD65+		41.6	F	3.0	6.9
18	HLAa: 0301:0501:0201 (DR3) HLAb: 0401:0301:0302 (DR4)	GAD65+		8.9	F	2.9	9.1
19	HLAa: 0404:0301:0302 (DR4) HLAb: 0301:0501:0201 (DR3)	GAD65+ ICA+		6.4	F	3.3	8.4
20	HLAa: 0301:0501:0201 (DR3) HLAb: 0101:0101:0501	GAD65+ ICA+		24.7	M	3.3	7.5
		**Average ± SE:**		**21.7 ± 3.6**		**3.2±0.2**	**8.4 ± 0.2**
**T1D subjects**							
**ID**			Serum AA*	**Age**	**Sex**	**Years after Onset**	**RNA RIN**
1	HLAa: 1301:0103:0608 HLAb: 0901:0301:0303		GAD65+ ICA512+ mIAA+ ICA+	23.2	M	2.3	7.4
2	HLAa: 0401:0301:0302 (DR4) HLAb: 0103:0101:0501		GAD65+ ICA512+ mIAA+	23.8	F	2.3	8.4
3	HLAa: 0901:0301:0303 HLAb: 1201:0501:0301		GAD65+ mIAAA+	26.8	F	2.3	7.8
4	HLAa: 0404:0301:0302 (DR4) HLAb: 0404:0301:0302 (DR4)		ICA512+ mIAA+ ICA+	13.1	F	2.3	8.5
5	HLAa: 0404:0301:0302 (DR4) HLAb: 0405:0301:0201 (DR4)		GAD65+ ICA512+ mIAA+	40.8	M	2.2	8.6
6	HLAa: 0401:0301:0302 (DR4) HLAb: 0101:0101:0501		GAD65+ mIAA+	18.2	F	2.3	8.2
7	HLAa: 0401:0301:0302 (DR4) HLAb: 0301:0501:0201 (DR3)		GAD65+ ICA512+ mIAA+	18.6	M	2.2	7.6
8	HLAa: 0301:0501:0201 (DR3) HLAb: 1601:0102:0502		GAD65+ mIAA+	14.7	F	2.2	7.6
9	HLAa: 0401:0301:0302 (DR4) HLAb: 0301:0501:0201 (DR3)		ICA512+ mIAA+ ICA+	16.7	F	2.2	7.3
10	HLAa: 0401:0301:0302 (DR4) HLAb: 0301:0501:0201 (DR3)		GAD65+ ICA512+ mIAA+	16.2	M	2.3	8.3
			**Average ± SE:**	**20.7 ± 2.5**		**2.8 ± 0.2**	**8.2 ± 0.2**

^*^HLAa and HLAb, HLA Haplotype α and HLA Haplotype β shown as DRB1:DQA1:DQB1. ^†^GAD65 - GAD65 standard (TN local) assay; Positive >0.032, ICA512 - ICA512/IA2 standard (TN local) assay; Positive >0.049, GAD65H - GAD65 harmonized assay; Positive >20, IA-2H - ICA512/IA2 harmonized assay; Positive >5, MIAA - mIAA; Positive >.01, ICA - Islet Cell Autantibody; Positive ≥10. Serum autoantibody information were provided by TrialNet.

### Animals

Female NOD/LtJ (NOD) and NOD.B10Sn-*H2^b^*/J (NOD.B10) mice were purchased from Jackson Laboratories (Bar Harbor, ME). Animals were maintained under pathogen-free conditions at the Stanford School of Medicine Animal facility, according to institutional guidelines under approved protocols. Pancreas tissues were isolated from 12-week-old NOD and NOD.B10 mice (8 animals per group) and immediately homogenized in Trizol reagent on ice, and stored at -80°C. RNA was extracted as described below. See **Supplementary Figure 1** for blood glucose levels, weight of mice, and bioanalyzer traces of RNA samples.

### RNA Extraction From Pancreas Tissue

**Figure 1:**
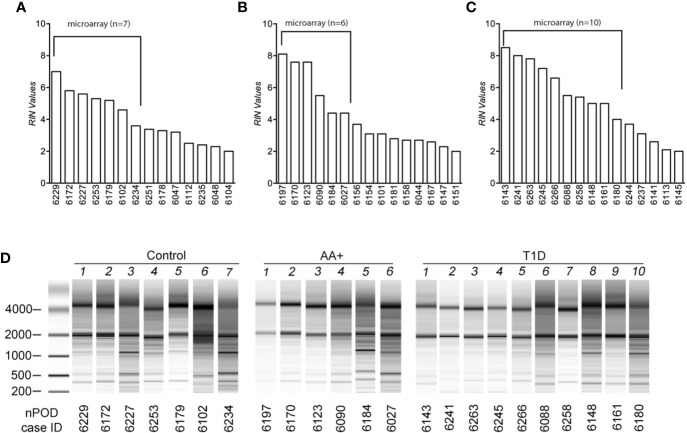
Assessment of RNA quality of samples extracted from human pancreatic tissues. RNA was isolated from pancreatic tissues of controls **(A)**, AA+ individuals **(B)**, and T1D patients **(C)** obtained from nPOD, and bioanalyzer analysis was performed to assess RNA quality. RIN (RNA integrity numbers) values **(A–C)** and bioanalyzer traces **(D)** are shown. The samples with the highest RIN values were used for microarray analysis (as indicated).

Total RNA was extracted using Trizol reagent and the Qiagen RNeasy mini kit, as previously described (28, 29). For human pancreas tissues, RNA was extracted from at least 14 samples per group (controls, AA+ and T1D). Pancreatic samples were thawed on ice, removed from RNAlater, and homogenized in Trizol reagent (100 mg tissue/ml Trizol) on ice. For human and NOD mouse samples, RNA was extracted from 1 ml of the tissue/Trizol homogenate. 0.2 ml chloroform was added to the homogenate, shaken vigorously and centrifuged at 12,000*xg* for 15 min at 4°C. The aqueous phase was removed, mixed with an equal volume of 70% ethanol and bound to a Qiagen RNeasy mini-column. The column was washed and RNA was eluted according to manufacturer’s instructions. Total RNA concentrations were measured by Nanodrop and quality was determined by Bioanalyzer analysis (Agilent Technologies, Santa Clara, CA). Bioanalyzer traces for human and mouse samples are shown in **Figure 1** and **Supplementary Figure 1**, respectively. The human samples with the highest RNA integrity numbers (RIN; n≥6 per group) were selected for gene expression analysis studies.

### Microarray Analysis

#### Human Samples

One-color microarrays were performed by the Stanford Human Immune Monitoring Center to measure gene expression in individual pancreas samples of seven controls, six AA+, and 10 T1D patients (**Table 1**) using the SurePrint G3 Human GE 8x60K microarray kit (Agilent Technologies). One microgram of total RNA was labeled with Cy3 using the low RNA input fluorescence linear amplification kit (Agilent Technologies). Hybridization was performed using the gene expression hybridization kit (Agilent Technologies) and the microarray hybridization chamber (Agilent Technologies), according to the manufacturer. Microarray chips were scanned using the DNA microarray scanner (Agilent Technologies) and data were processed with Feature Extraction Software (Agilent Technologies), and analyzed using GeneSpring GX 12.6.1 Software (Agilent Technologies). All microarray data have been submitted to the Gene Expression Omnibus (GEO) Database at NCBI (GEO series accession number: GSE72492), and are accessible at: http://www.ncbi.nlm.nih.gov/geo/query/acc.cgi?acc=GSE72492. Samples were filtered for entities that were detected in at least six out of 23 samples, and entities with annotated gene symbols. Because of the gender imbalance between the groups, genes that were significantly changed by ≥5 fold between male and female subjects were removed from the analysis (**Supplementary Table 1**). A final list of 29,438 entities was used for further analysis. Moderated T-tests were performed to identify differentially expressed genes (p<0.05), and pathway analysis of differentially expressed genes was performed using Ingenuity Pathway Analysis (IPA; Qiagen Inc; https://www.qiagenbioinformatics.com/products/ingenuitypathway-analysis).

#### Mouse Samples

Two-color microarrays were performed to measure gene expression in the pancreas of individual 12-week-old NOD mice against a pool of age-matched NOD.B10 mice (n=8 animals per group) using the Whole Mouse Genome Microarray Kit, 4×44K 2-color arrays (Agilent Technologies) as previously described (29). Data were analyzed using GeneSpring (version 12.6.1, Agilent Technologies). Samples were filtered for entities with annotated gene symbols that are expressed (minimum raw expression ≥ 25) in at least four of the eight NOD samples, have equivalent human homologues, and are expressed in the human pancreas samples. The resultant 25,283 entities were analyzed. Data are available at GEO (Series accession number: GSE154739, http://www.ncbi.nlm.nih.gov/geo/query/acc.cgi?acc=GSE154739). T-test and Benjamini-Hochberg multiple testing correction were performed to identify differentially expressed genes in the pancreata of NOD vs. NOD.B10 mice with a fold change ≥2-fold (corrected *p<0.01*).

### Validation by QPCR

We identified a number of biologically relevant genes among those that were changed in the pancreata of AA+ individuals vs. controls. These include genes that have previously been associated with T1D, T2D, metabolism or obesity, and genes with biological functions that may be involved in the pathogenesis of T1D. These genes and their significance are listed in **Table 3**.

**Table 3:** Biologically relevant genes that are significantly changed in the pancreata of AA+ vs. control (Microarray experiments).

Genes associated with T1D, T2D, metabolism, or obesity				
Gene	Description	Significance	Reference	
***CADM2***	cell adhesion molecule 2	SNP associated with risk of obesity, regulates insulin sensitivity	Speliotes et al. (30); Dorajoo, 2012; Rathjen et al. (31); Yan, 2018	
***BCL2L15***	BCL2-like 15	Gene located in a T1D-associated loci.	T1Dbase.org	
***ERAP1***	endoplasmic reticulum aminopeptidase 1	Gene located in a T1D-associated loci.	Fung et al. (32)	
***ETV5***	ets variant 5	SNP located near ETV5 gene linked to obesity.	Thorleifsson et al. (33)	
***KANK1***	KN motif and ankyrin repeat domains 1	Variants associated with fasting proinsulin & insulinogenic index	Huyghe et al. (34)	
***SIM1***	single-minded family bHLH transcription factor 1	Gene in an obesity-associated loci.	Meyre et al. (35)	
***PLXNA4***	plexin A4	Gene associated with T2D.	Saxena et al. (36)	
***TRPM5***	transient receptor potential cation channel, subfamily M, member 5	TRPM5 variants associated with pre-diabetic phenotypes. Regulates insulin secretion and loss of expression leads to a pre-diabetic phenotype.	Brixel et al. (37); Ketterer et al. (38)	
***ABCB9***	ATP-binding cassette, sub-family B, member 9	Gene variant that confers susceptibility for T2D.	Harder et al. (39)	
**Biologically relevant to pathogenesis of T1D – Genes upregulated in AA+**				
***PGC***	Progastricsin (pepsinogen C)	Expressed in human islets and is converted to the proteolytic enzyme pepsin in low pH.		Hassan et al. (40)
***CEACAM6***	carcinoembryonic antigen-related cell adhesion molecule 6	Involved in insulin homeostatsis and T cell proliferation, and serves as receptor for host-specific viruses and bacteria.		Kuespert, 2006
***CALB1***	calbindin 1, 28kDa	Overexpression reduces glucose-stimulated insulin secretion by modulating calcium influx.		Lee et al. (41)
***S100B***	S100 calcium binding protein B	An inflammatory protein that serves as a ligand for the receptor of advanced glycation end products that are involved in the development of pathogenic T cells and apoptosis of beta cells.		Chen et al. (42)
***SMPD3***	sphingomyelin phosphodiesterase 3,neutral membrane	Can mediate cellular responses to IL-1b and TNFa.		Rutkute et al. (43)
***IL33***	interleukin 33	May promote the development of inflammatory autoimmune T cells in experimental models of T1D.		Milovanovic et al. (44)
***ETV5***	ets variant 5	Controls differentiation of TH17 cells.		Pham et al. (45)
**Biologically relevant to pathogenesis of T1D—Genes downregulate in AA+**				
***CRH***	corticotropin releasing hormone	expressed in beta cells. Plays a role in insulin secretion and beta cell proliferation.		Kanno et al. (46); Huising et al. (47)
***PDK4***	pyruvate dehydrogenase kinase, isozyme 4	A key modulator of glucose homeostasis. Regulates expression of the transcription factor Ptf1a.		Dateki et al. (48); Zhang, 2014
***ANGPTL4***	angiopoietin-like 4	Involved in glucose homeostasis, lipid metabolism, and insulin sensitivity. Regulates islet morphology.		Xu et al. (49); Kim et al. (50)
***SCIMP***	SLP adaptor and CSK interacting membrane protein	Expressed on B cells and involved in signal transduction after MHCII stimulation.		Draber et al. (51)
***SEZ6L***	seizure related 6 homolog-like	A substrate for BACE2, an inhibitor of beta cell proliferation.		Stutzer et al. (52)
***ICOSLG***	inducible T-cell co-stimulator ligand	Provides co-stimulation through ICOS, and may be involved in the expansion of activated Tregs		Martin-Orozco et al. (53)
***PLD1***	phospholipase D1, phosphatidylcholine-specific	Phosphatidic acid formation on the graunule membrane by PLD1 is essential for glucose-stimulated insulin secretion		Ma et al. (54)
***TRPM5***	transient receptor potential cation channel, subfamily M, member 5	TRPM5 variants associated with pre-diabetic phenotypes. Regulates insulin secretion and loss of expression leads to a pre-diabetic phenotype.		Brixel et al. (37); Ketterer et al. (38)
***GNLY***	granulysin	An antimicrobial peptide. Reduced *GNLY* expression has been observed in the PBMCs of T1D patients		Jin et al. (55)
***CRTAM***	cytotoxic and regulatory T cell molecule	Involved in establishing late phase T cell polarity, and allows increased production of IFNγ and IL22.		Yeh et al. (56)
***CD180***	CD180 molecule (RP105)	An important modulator of Toll-like receptor 4 signaling that is involved in activating innate and adaptive immune responses.		Divanovic et al. (57)
***FCGR2B***	Fc fragment of IgG, low affinity IIb, receptor	Impaired FCGR2B function leads to abberant B cell activation and the development of autoimmunity. Reduced *Fcgr2b* linked to autoimmune susceptibility in NOD mice.		Jiang et al. (26); Xiu et al. (27); Anania et al. (20)
***CLEC4D***	C-type lectin domain family 4, member D	Plays a non-redundant role in protecting against anti-mycobacterial and fungal infection.		Wilson et al. (58)
***IL4R***	interleukin 4 receptor	IL4 can prevent autoimmune diabetes in rodent models of T1D.		Ko et al. (59)
***RGS16***	regulator of G-protein signaling 16	Key factor in G-protein mediated activation of lymphocytes. Plays a role in beta-cell development, proliferation, and insulin secretion.		Villasenor et al. (60); Vivot et al. (61)

The following references are included in **Table 3** (26, 31–37, 39–46, 48, 50–59, 61).

QPCR was performed to validate differential gene expression. cDNA was synthesized and QPCR was performed as previously described (28, 29). Briefly, first strand cDNA was generated from 1 μg of total RNA using Superscript III (Invitrogen) and random hexamers according to manufacturer’s instructions. QPCR was performed to measure human *INS*, *BCL2L15, ERAP1, CADM2, ERAP1, ETV5, KANK1, SIM1, ABCB9, PGC, CEACAM6, CALB1, S100B, SMPD3, IL33, SCIMP, SEZ6L, PLD1, GNLY, CRTAM, CD180, IL4R, PLXNA4, PDK4, FCGR2B, TRPM5, CRH, ANGPTL4, CLEC4D, RGS16, ICOSLG, CD19, CD20, CD11C*, and housekeeping genes *GAPDH, ACTB, B2M*, and 18S rRNA using commercially available Taqman gene expression assays (Applied Biosystems). cDNA was preamplified using the Taqman PreAmp Mastermix (Applied Biosystems) prior to QPCR for measurements that initially gave threshold cycle (Ct) values of >30. The 7900HT Fast Real Time PCR System (Applied Biosystems) and the Taqman Gene Expression Mastermix (Applied Biosystems) were used, according to manufacturer’s instructions. The comparative Ct method for relative quantification (ΔΔCt) was used. QPCR was also performed to measure the expression of *FCGR2B, CD19, CD20, CD11C*, and *ACTB* in whole blood RNA samples obtained from TrialNet. For these experiments, cDNA was synthesized (from 100 ng of total RNA) and QPCR was performed to described above.

### Statistical Analysis

Statistical analyses for QPCR experiments were performed using the two-tailed Mann-Whitney test (Prism 8, GraphPad Software Inc. San Diego, CA). A P-value of ≤0.05 was considered significant.

## Results

### Gene Expression Analysis in Human Pancreata Samples

**Figure 2:**
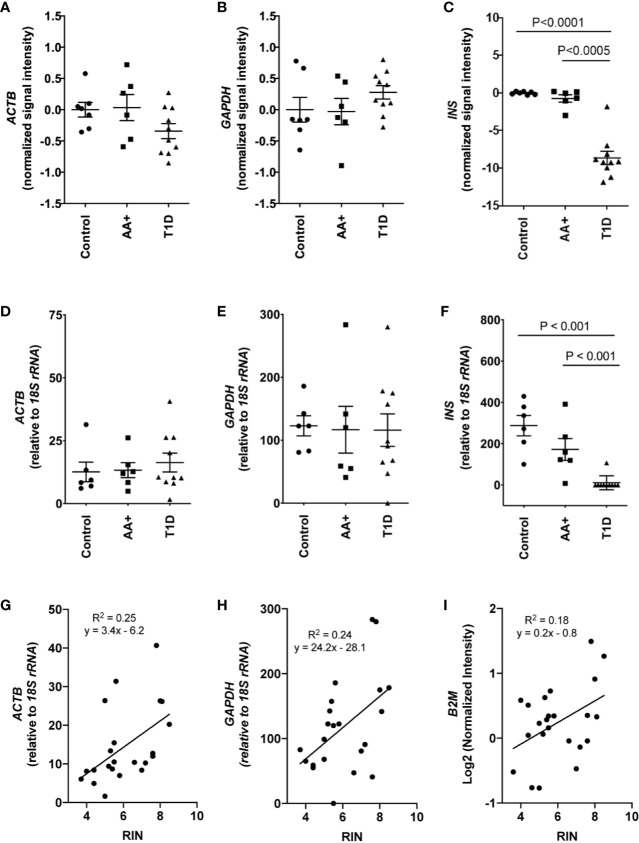
Comparison of house-keeping gene and *INS* expression in control, AA+ and T1D samples. **(A–C)** Microarray data showing the normalized signal intensity for *ACTB* **(A)**, *GAPDH* **(B)** and *INS* **(C)** in individual control, AA+ and T1D samples. No difference in *ACTB* and *GAPDH* expression was observed between groups. *INS* expression was significantly lower in T1D patients. **(D–F)** QPCR analysis showing similar data for *ACTB* **(D)**, *GAPDH* **(E)**, and *INS* **(F)** expression. **(G–I)** Graphs showing the expression of house-keeping genes *ACTB, GAPDH*, and *B2M* against RIN values. Statistical analysis was performed using the Moderated T-test (panel A-C) or the two-tailed Mann-Whitney test (Panel **D–F**).

Total RNA was initially isolated from ≥14 subjects per group (healthy controls, AA+ and T1D subjects). Bioanalyzer analysis showed considerable RNA degradation in some samples, with RIN (RNA integrity number) values ranging from 2.0 to 8.5 (**Table 1**, **Figure 1**). The majority of samples had a RIN ≥5, and samples with RIN values of ≥3.7 were used for microarray analysis (n=7 controls, 6 AA+, and 10 T1D samples). Microarray and QPCR results showed that the expression of housekeeping genes *ACTB, GAPDH* did not significantly differ between groups and did not strongly correlate with RIN values (**Figure 2**). As expected, *INS* (Insulin) expression was found to be significantly reduced in the pancreata of T1D patients compared to controls, and pre-diabetic AA+ individuals (**Figure 2**).

Microarray analysis was performed on 29,168 entities representing 20,813 genes that were expressed in at least six of the 23 human pancreata. Because of the gender imbalance between groups, we eliminated genes from our analysis that were significantly changed by >5-fold in male vs. female subjects (**Supplementary Table 1**). The expression of certain genes, however, may still be influenced by age or sex.

**Figure 3:**
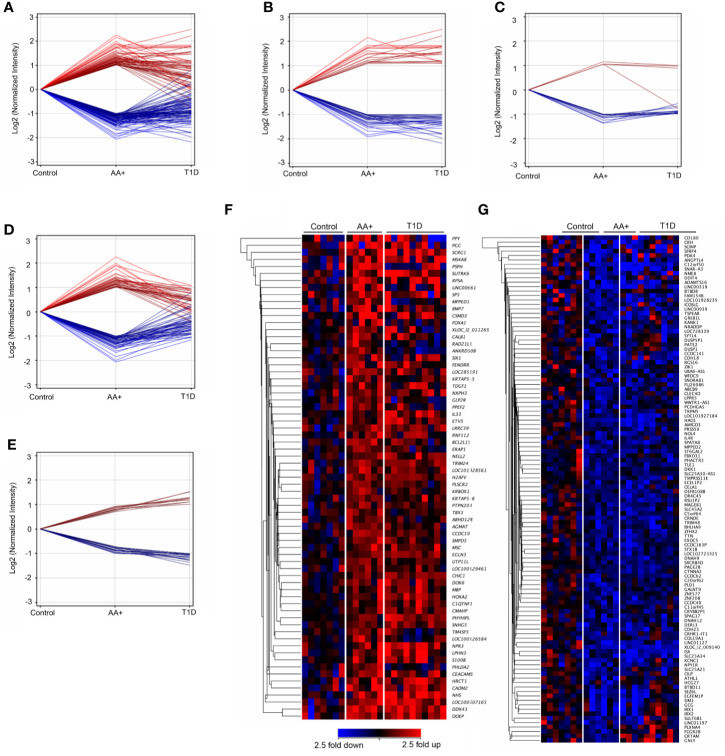
Differentially expressed genes in the pancreata of AA+ individuals compared to controls. 155 genes (59 upregulated and 96 downregulated) were significantly changed by at least 2-fold in the pancreata of AA+ individuals vs. controls **(A)**. Among these, 48 were changed by >2-fold **(B)**, 19 were changed by <2-fold **(C)**, and 88 were unchanged **(D)** in the pancreata of T1D patients vs. controls. An additional 28 genes were significantly changed by 1.5 to 2-fold in AA+ vs. controls and >2-fold in T1D vs. controls **(E)**. Gene expression heatmaps of the upregulated **(F)** and downregulated **(G)** genes from panels A and E are shown. These genes were further analyzed for biological function (See **Supplementary Table 2** and **Table 3**).

We showed that 155 genes (59 upregulated and 96 downregulated) were changed by ≥2-fold in the pancreata of AA+ compared to controls (**Figure 3A**; **Supplementary Table 2**), and 645 genes (252 genes upregulated and 395 downregulated) were changed by ≥2-fold in the pancreata of T1D patients compared to controls (**Supplementary Table 3**). Among the 155 genes changed by ≥2-fold in the pancreata of AA+ individuals (**Figure 3A**), 48 genes were similarly and significantly changed by ≥2-fold in T1D patients (**Figure 3B**), 19 were significantly changed by <2-fold in T1D patients (**Figure 3C**), and 88 were not significantly changed in T1D patients (**Figure 3D**). Another 28 genes were found to be significantly changed by 1.5 to 2-fold in AA+ vs. controls, but >2-fold in T1D vs. controls (**Figure 3E**). These data indicate that 107 genes may be transiently changed after seroconversion, but no longer changed after the establishment of hyperglycemia, while 76 genes may be changed after seroconversion and during T1D. Heatmaps of gene expression for the 183 genes (represented in **Figures 3A, E**) are shown in **Figure 3F** (upregulated genes) and **Figure 3G** (downregulated genes).

### Pathway Analysis of Differentially Expressed Genes in At-Risk AA+ vs. Controls

IPA pathway analysis was performed on the 183 genes that are differentially expressed in the pancreata of AA+ individuals compared to controls. A significant association of genes with various immune response functions was observed (**Table 4A**), with the strongest association seen with “function of helper T lymphocytes” (*p = 1.93x10^-6^*). Upstream regulator analysis using IPA identified several cytokines including IL-1β that may drive the observed changes in gene expression (**Table 4B**). The top upstream regulator was found to be CNR1 (canniboid receptor 1, also known as the CB1 receptor; *p*<2.55 x 10^-5^), a receptor that has been linked to β-cell dysfunction and islet inflammation (62).

**Table 4A:** Immune functions associated with differentially expressed genes in AA+ vs. control identified by IPA.

Diseases or Functions Annotation	p-Value*	Genes
function of helper T lymphocytes	1.93E-06	*CRTAM, DUSP1, ICOSLG, IL33, IL4R, NPY1R, RGS16*
proliferation of B-1 lymphocytes	1.71E-04	*FCGR2B, IL33, IL4R*
function of Th1 cells	2.89E-04	*CRTAM, DUSP1, NPY1R, RGS16*
function of Th2 cells	3.69E-04	*ICOSLG, IL33, IL4R, RGS16*
quantity of IgG2a	9.03E-04	*CD180, FCGR2B, ICOSLG, IL4R, NPY1R*
degranulation of leukocytes	9.07E-04	*FCGR2B, PLD1, PSPH, PTPN20B*
accumulation of Th2 cells	1.30E-03	*IL33, RGS16*
function of T lymphocytes	1.41E-03	*CRTAM, DUSP1, ERAP1, ICOSLG, IL33, IL4R, NPY1R, RGS16*
quantity of IgG2b	1.75E-03	*CD180, FCGR2B, ICOSLG, NPY1R*
induction of leukocytes	2.44E-03	*CRH, IL33, IL4R, MBP*
degranulation of mast cells	3.14E-03	*CRH, DUSP1, FCGR2B, IL4R, PLD1*
quantity of interleukin	3.97E-03	*CLEC4D, CRH, DUSP1, IL4R, NPY1R*
stimulation of mast cells	4.12E-03	*CRH, IL33*
Th2 immune response	4.24E-03	*ICOSLG, IL33, IL4R*
function of immune system	4.68E-03	*CLEC4D, FCGR2B, ICOSLG, IL4R, NPY1R*
activation of basophils	6.07E-03	*FCGR2B, IL33*
function of blood cells	6.67E-03	*CRH, CRTAM, DUSP1, ERAP1, FCGR2B, ICOSLG, IL33, IL4R, NPY1R, PLD1, RGS16, TRPM5*
induction of helper T cells	6.80E-03	*IL33, MBP*
accumulation of T lymphocytes	6.82E-03	*DUSP1, ICOSLG, IL33, RGS16*
induction of lymphocytes	7.28E-03	*CRH, IL33, MBP*
activation of blood cells	8.05E-03	*C1QTNF1, CD180, CEACAM6, CRH, CRTAM, DUSP1, ERAP1, FCGR2B, GNLY, ICOSLG, IL33, IL4R, MBP, NPY1R*
infiltration of cells	8.45E-03	*CRH, DUSP1, EGLN3, FCGR2B, IL33, IL4R, IRX2, MBP, S100B*
stimulation of phagocytes	8.78E-03	*CRH, IL33, IL4R*
function of leukocytes	8.83E-03	*CRH, CRTAM, DUSP1, ERAP1, FCGR2B, ICOSLG, IL33, IL4R, NPY1R, RGS16, TRPM5*
quantity of IgG	9.17E-03	*CD180, CLEC4D, FCGR2B, ICOSLG, IL4R, NPY1R*
quantity of lymph node cells	1.01E-02	*ICOSLG, IL33*
activation of leukocytes	1.08E-02	*CD180, CEACAM6, CRH, CRTAM, DUSP1, ERAP1, FCGR2B, GNLY, ICOSLG, IL33, IL4R, MBP, NPY1R*
stimulation of leukocytes	1.08E-02	*CRH, ICOSLG, IL33, IL4R, MBP*
quantity of immunoglobulin	1.09E-02	*CD180, CLEC4D, FCGR2B, ICOSLG, IL33, IL4R, NPY1R*
quantity of dendritic cells	1.25E-02	*CLEC4D, FCGR2B, GNLY, TRPM5*
hypersensitive reaction	1.26E-02	*CLEC4D, DUSP1, FCGR2B, ICOSLG, IL33, IL4R, MBP, NPY1R, PDK4*
stimulation of lymphocytes	1.35E-02	*CRH, ICOSLG, IL33, MBP*
quantity of transitional B cells	1.61E-02	*CD180, NPY1R*
quantity of lymph follicle	1.65E-02	*CD180, FCGR2B, ICOSLG, NPY1R*
inflammation of pancreas	1.72E-02	*FCGR2B, IL33*
maturation of bone marrow-derived dendritic cells	1.72E-02	*FCGR2B, NPY1R*
infiltration of leukocytes	1.77E-02	*CRH, DUSP1, EGLN3, FCGR2B, IL33, IL4R, IRX2, S100B*
delayed hypersensitive reaction	1.77E-02	*CLEC4D, FCGR2B, IL4R, NPY1R*
quantity of cytokine	1.79E-02	*CLEC4D, CRH, DUSP1, IL4R, NPY1R, SIM1*
development of Th2 cells	1.83E-02	*ICOSLG, IL4R*
proliferation of mast cells	1.83E-02	*FCGR2B, IL33*
quantity of IgA	1.88E-02	*FCGR2B, ICOSLG, NPY1R*
activation of lymphocytes	1.98E-02	*CD180, CEACAM6, CRTAM, DUSP1, FCGR2B, ICOSLG, IL33, MBP, NPY1R*

*Performed using Fisher’s exact test to measure likelihood that the association between the dysregulated gene set and a related function is due to random association.

**Table 4B:** Upstream regulators of differentially expressed genes in AA+ vs. control identified by IPA analysis.

Upstream Regulator	p-Value	Targets
IL-1β	3.42E-03	*ANGPTL4, CALB1, CRH, DDIT4, DUSP1, EGLN3, FCGR2B, ICOSLG, IL33, PLD1, RGS16, RPSA, S100B*
IL-10	2.74E-02	*DUSP1, FCGR2B, IL33, IL4R, RGS16, S100B*
TNFSF13B (BAFF)	4.10E-02	*FCGR2B, ICOSLG*
TNF	2.15E-01	*ANGPTL4, CRH, DKK1, DUSP1, FCGR2B, ICOSLG, IL33, IL4R, MBP, MSC, RGS16, RPSA, UTP11L*
IL-6	4.66E-01	*CRH, DUSP1, GCG, ICOSLG, IL4R*

### QPCR Validation of Biologically Relevant Genes That Are Changed in AA+ Individuals

**Figure 4:**
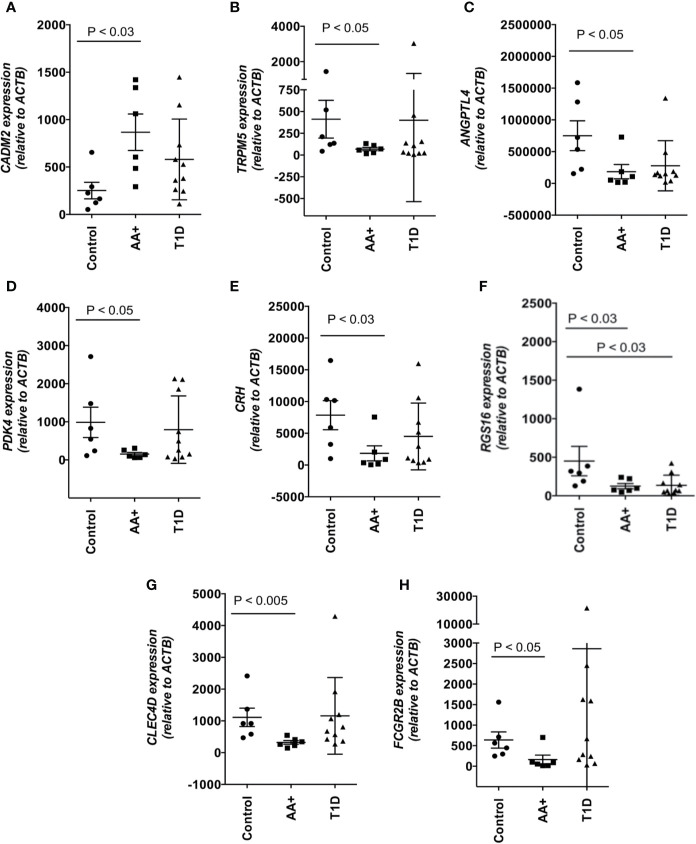
Changes in the expression of 8 disease-relevant genes were verified by QPCR analysis. A significant upregulation of *CADM2* **(A)**, and downregulation of *TRPM5* **(B)**, *ANGPTL4* **(C)**, *PDK4* **(D)**, *CRH* **(E)**, *RGS16* **(F)**, *CLEC4D* **(G)**, and *FCGR2B* **(H)** was verified in the pancreata of AA+ vs. control individuals by QPCR analysis. Statistical analysis was performed using the two-tailed Mann-Whitney test. (See **Supplementary Figure 2** for complete set of QPCR data of all genes shown in **Table 3**).

Among the 183 genes that are significantly changed in the pancreata of AA+ individuals vs. controls, we identified 9 genes that are located in genetic loci that have previously been associated with T1D, T2D, metabolism or obesity, by GWAS studies. In addition, we identified another seven upregulated genes, and 15 downregulated genes, that have functions biologically relevant for disease pathogenesis (**Table 3**). The expression of these genes was verified by QPCR analysis. We were able to validate changes in eight out of 29 genes examined (**Figure 4**, **Supplementary Figure 2**, and **Table 5**). A significant upregulation of *CADM2*, and downregulation of *TRPM5, CRH*, *PDK4, ANGPTL4, CLEC4D, RGS16*, and *FCGR2B* was confirmed in the pancreata of AA+ vs. controls. Fold-change differences were higher when gene expression was measured by QPCR vs. microarray analysis. The QPCR cycle threshold (Ct) values for these genes are shown in **Supplementary Table 5**. RNA RIN values of the samples did not appear to influence the expression of these 8 genes when measured by microarray or QPCR analysis (**Supplementary Figures 3** and **4**).

**Table 5:** Microarray and QPCR data for biologically relevant genes that are differentially expressed in the pancreata of AA+ and T1D patients vs. controls.

GWAS genes changed in AA+ vs. controls				
Gene	Microarray		QPCR	
	Fold change AA+ vs. Controls	Fold change T1D vs. Controls	Fold change AA+ vs. Controls	Fold change T1D vs. Controls
***CADM2***	3.1*	2.3*	3.7*	2.3
***BCL2L15***	2.7*	1.4	1.3	1.3
***ERAP1***	2.1*	1.1	1.3	1.2
***ETV5***	1.8*	2.4*	1.4	5.0
***KANK1***	-2.5*	-1.4	-1.5	-1.0
***SIM1***	-2.4*	-2.2*	-3.2	-1.6
***PLXNA4***	-2.2*	1.2	-1.2	-1.1
***TRPM5***	-2.2*	-1.8	-6.0*	-1.0
***ABCB9***	-2.2*	-2.1*	-1.3	-1.4
**Biologically relevant genes: upregulated in AA+ vs. controls**				
**Gene**	**Microarray**		**QPCR**	
	**Fold change** **AA+ vs. Controls**	**Fold change** **T1D vs. Controls**	**Fold change** **AA+ vs. Controls**	**Fold change** **T1D vs. Controls**
***PGC***	4.8*	1.8	15.8	3.5
***CEACAM6***	2.9*	1.9	2.6	3.9
***CALB1***	2.6*	1.7	3.2	4.4
***S100B***	2.5*	2.7*	1.4	2.1
***SMPD3***	2.3*	1.7	-1.2	-1.4
***ETV5***	1.8*	2.4*	1.4	5.0
***IL33***	1.7*	2.9*	-1.6	3.1
**Biologically relevant genes: downregulated in AA+ vs. controls**				
**Gene**	**Microarray**		**QPCR**	
	**Fold change** **AA+ vs. Controls**	**Fold change** **T1D vs. Controls**	**Fold change** **AA+ vs. Controls**	**Fold change** **T1D vs. Controls**
***CRH***	-4.2*	-2.2	-4.3*	-1.7
***PDK4***	-4.0*	-1.6	-6.4 *	-1.2
***ANGPTL4***	-3.6*	-2.6*	-4.1*	-2.7
***SCIMP***	-3.6*	-2.0	-2.5	1.3
***SEZ6L***	-3.0*	-1.9	-5.0	-2.6
***ICOSLG***	-2.6*	-1.6	-1.4	1.3
***PLD1***	-2.5*	-1.4	-1.7	-1.1
***TRPM5***	-2.2*	-1.6	-6.0*	-1.0
***GNLY***	-2.2*	1.8	1.1	10.5
***CRTAM***	-2.2*	1.4	-3.7	-1.1
***CD180***	-2.2*	1.1	-2.1	-1.3
***FCGR2B***	-2.1*	1.2	-4.0 *	4.5
***CLEC4D***	-2.1*	-1.3	-3.4*	1.0
***IL4R***	-2.0*	-1.9*	-1.7	1.4
***RGS16***	-1.7*	-2.4*	-3.5*	-3.3*

*P < 0.05 for AA+ vs. control or T1D vs. control. See Methods for statistical analysis.

Our inability to validate additional genes may be due to inherent differences in the quantification methods used. QPCR data is normalized to house-keeping gene expression and may be affected by the choice of house-keeping gene. The Taqman assays used for QPCR may also contain primers and probes that target a different region of the gene compared to the probes on the microarray. Thus, differential expression of gene transcripts or alternative splicing of genes could result in differences between microarray and QPCR data.

**Figure 5:**
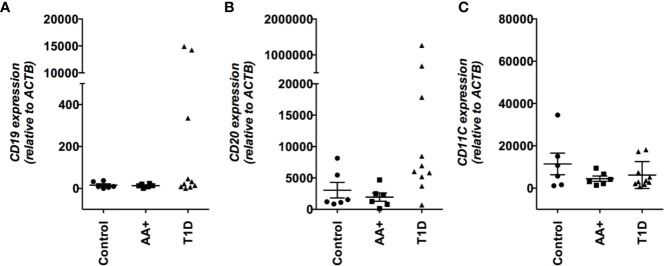
Expression *CD19, CD20*, and *CD11c* in the pancreata of AA+ and T1D patients compared to controls. Expression levels were measured by QPCR and statistical analysis was performed using the two-tailed Mann Whitney test.

Among the genes that we validated by QPCR, the change in *FCGR2B* expression is particularly interesting. This gene has been shown to play a role in multiple autoimmune diseases and a role in the development of NOD disease (26). *FCGR2B* encodes the inhibitory CD32B receptor that is mainly expressed in B-cells (20). To examine if the change in *FCGR2B* expression may simply reflect a change in the abundance of immune cells, we measured *CD19, CD20* and *CD11C* expression, and found no significant difference in expression between controls, AA+ and T1D patients (**Figure 5**).

### Comparison of Differentially Expressed Genes in At-Risk AA+ Individuals and Pre-Diabetic NOD Mice

**Figure 6:**
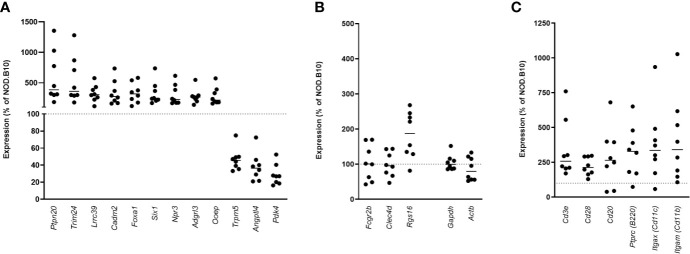
Gene expression in the pancreata of pre-diabetic 12-week-old NOD mice compared to AA+ individuals. 12 genes were found to be similarly changed in the pre-diabetic pancreata of 12-week-old NOD vs. NOD.B10 control mice and the pancreata of AA+ individuals vs. controls **(A)** Surprisingly, 4 of these genes were among the biologically relevant genes that we verified by QPCR (**Figure 4**). *Fcgr2b, Clec4d*, and *Rgs16* were not found to be reduced in the pancreata of NOD mice **(B)**, likely due to the high level of insulitis. Abundant expression of various immune cell markers was observed in the NOD pancreas **(C)**.

To determine if similar genes are altered in the pancreata of AA+ individuals and pre-diabetic NOD mice, we performed microarray analysis on pancreas samples of 12-week-old NOD mice compared to age-matched congenic non-diabetes-prone NOD.B10 mice. Female NOD mice develop insulitic lesions starting at 4 weeks of age, and the onset of destructive insulitis occurs at 12 weeks age, followed by hyperglycemia at ~16 weeks of age (13). For these experiments, pancreatic tissues of 12-week-old NOD and NOD.B10 mice were immediately isolated and homogenized in Trizol reagent. RNA prepared from these samples showed little RNA degradation, with RIN values exceeding 8 for all samples (**Figure S1**). 25,283 entities representing 15,119 genes expressed in NOD pancreas samples overlap with genes on the human microarray. Among these, 2,043 entities representing 1880 genes were differentially expressed by at least 2-fold (*p<0.01*) in the pancreata of NOD vs. NOD.B10 mice. Only 12 genes were found to be similarly changed in the pancreata of NOD mice and human AA+ individuals (**Figure 6A**). Interestingly 4 of the 12 genes were among those that we successfully validated by QPCR (**Figure 4**). We showed that *Cadm2* was significantly increased while *Trpm5, Pdk4* and *Angptl4* were significantly reduced in the pancreata of NOD vs. NOD.B10 mice. No changes in housekeeping genes *Actb* and *Gapdh* were observed (**Figure 6A**). *Crh* was not present in the mouse microarray. The other three genes, *Fcgr2b, Clec4d*, and *Rgs16*, that were reduced in the pancreata of AA+ individuals, were not changed in the pancreata of NOD mice (**Figure 6B**). These genes are abundantly expressed on B-cells, T-cells, DCs, and/or macrophages (20, 63–65). Thus their expression in the pancreas is impacted by the presence of immune cells in the insulitic lesion of NOD mice. Accordingly, we found significantly elevated expression of various leukocyte markers including *Cd3e* and *CD28* (T-cell markers)*, Cd20* and *B220 (*B-cell markers), *Cd11c* (*Itgax*; dendritic cell marker), and *Cd11b* (*Itgam;* macrophage marker) in the pancreata of NOD vs. NOD.B10 mice (**Figure 6C**).

### *FCGR2B* Gene Expression in Whole Blood Samples

**Figure 7:**
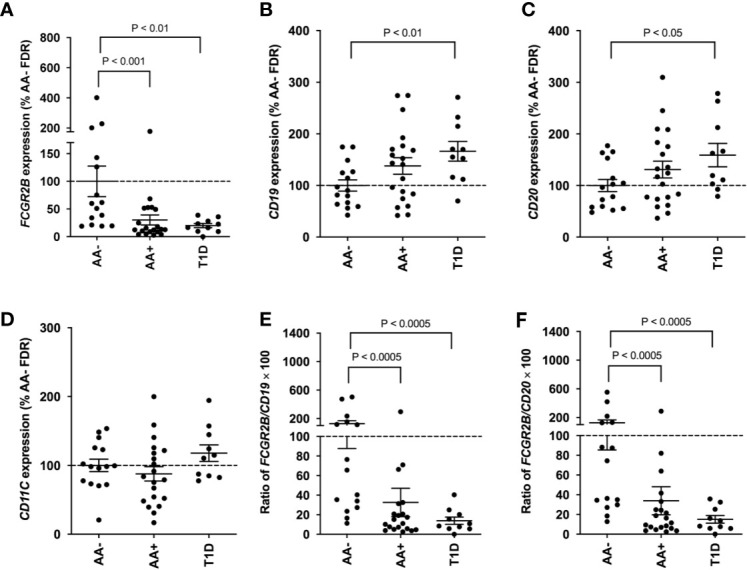
Loss of *FCGR2B* gene expression in the peripheral blood of AA+ FDRs and T1D patients. QPCR data showing *FCGR2B* **(A)**, *CD19* **(B)**, *CD20* **(C)**, and *CD11c* **(D)** expression in the whole blood of AA- FDRs, AA+ progressors, and T1D patients. *FCGR2B* expression is also shown relative to *CD19* **(E)** and *CD20* **(F)** expression for each individual. Statistical analysis was performed using the two-tailed Mann-Whitney test.

Since *FCGR2B* is highly expressed on B-cells, we asked whether the expression of this gene could serve as a potential biomarker of disease progression in peripheral blood cells. QPCR was performed to measure *FCGR2B* expression in whole blood samples of AA- first-degree relatives of T1D patients (AA- FDRs; n=15), AA+ FDRs who later progressed to hyperglycemia (AA+ progressors, n=20), and established T1D patients. Remarkably, we showed that *FCGR2B* expression was significantly lower in the whole blood of AA+ progressors and T1D patients compared to AA- FDRs (**Figure 7A**). *CD19, CD20*, and *CD11c* expression was not changed in AA+ progressors, while *CD19* and *CD20* expression was significantly elevated in peripheral blood cells of T1D patients vs. AA- FDRs (**Figures 7B–D**). When *FCGR2B* is expressed as a ratio of the B-cell markers *CD19* or *CD20*, the differences observed between AA+ progressors and T1D vs. AA- FDRs were more significant (**Figures 7E, F**). These data suggest that a loss of *FCGR2B* expression may occur in the peripheral blood, possibly in B-cells, during the development and progression of T1D.

## Discussion

A number of gene expression analysis studies have previously been performed to examine disease pathogenesis in T1D. The majority of studies, however, have been carried out using peripheral blood samples of AA+ at-risk and early onset T1D patients, while relatively few have been performed in the pancreata of these individuals, where insulitis, and islet destruction occur (66–72). This is partly due to the lack of pancreas samples available for study, and the difficulty in extracting high-quality RNA from these tissues. High levels of endogenous RNAses, DNases and proteases present in the pancreas result in the rapid autolysis of tissues following tissue damage, and lead to varying levels of RNA degradation (16, 73, 74). This is frequently observed in tissues collected post-mortem, when extensive tissue processing times are unavoidable. Pancreatic tissues collected from live individuals yield high quality RNA, but are difficult to obtain. In the Diabetes Virus Detection (DiViD) study, pancreatic biopsies were collected from live recent onset T1D patients, however, enrollment was halted when serious complications developed in 3 of the 6 individuals enrolled in the study (75, 76).

In this study, we utilized pancreatic tissue samples from JDRF nPOD for gene expression analysis. This repository contains one of the largest collections of tissues recovered from at-risk AA+ and T1D patients. Pancreatic tissues collected by nPOD are routinely minced and treated with RNAlater to increase RNA stability (73). The quality of the RNA extracted from pancreatic samples stored at nPOD has previously been assessed, and found to vary based on several factors including cause of death. Samples from head trauma victims had lower RIN values (18). This was also observed in our set of samples (**Table 1**). While appreciable levels of RNA degradation occurred in some of the samples, we were still able to identify a number of biologically relevant genes that were differentially expressed in AA+ and T1D patients compared to controls. Others have already shown that degraded RNA can be used successfully for transcriptome analysis and that the biological differences between groups outweigh the effects of RNA degradation on gene expression (19).

This study is the first to show that a distinct subset of genes is differentially expressed in the pancreata of at-risk AA+ individuals compared to T1D patients and to healthy controls. Of the 155 genes changed by >2-fold in the pancreata of AA+ individuals, only one-third remained changed in the pancreata of established T1D patients, reflecting the transient nature of events that are active during the early stages of disease development. After the establishment of hyperglycemia, the number of differentially expressed genes increased by ~4-fold to 645 genes. This includes genes such as *INS* that are lost as a result of β-cell death (**Figure 2**).

Changes in the gene expression profile of AA+ individuals reflect the activation of various immune pathways (**Table 4A**), and are consistent with the increased CD4+ and CD11c+ cell infiltration previously observed in the pancreata of AA+ patients (9). A number of cytokines including IL-1β, IL-10, TNSF13B (BAFF), TNF, and IL-6 were identified by IPA analysis to be upstream regulators of various differentially expressed genes (**Table 4B**). These cytokines have already been shown to play a role in the development of T1D (77–82).

To identify novel mechanisms that may contribute to disease pathogenesis, we focused on biologically relevant genes that were changed in the pancreata of AA+ individuals, and successfully validated the differential expression of 8 genes (*CADM2*, *TRPM5, PDK4, ANGPTL4, CRH*, *CLEC4D, RGS16*, and *FCGR2B*) by QPCR. We also performed gene expression analysis on 12-week-old NOD mice, during the onset of destructive insulitis, to identify genes or disease mechanisms that may be similarly changed in the pancreata of pre-diabetic NOD mice and “pre-diabetic” AA+ at-risk individuals. NOD disease shares many similarities with human T1D, however, there are striking differences in disease progression, especially in the pancreas where relatively mild insulitis is observed in humans, and large insulitic lesions and highly infiltrated islets are observed in NOD mice (83). As a result, the most differentially expressed genes observed in the pancreata of pre-diabetic NOD mice vs. NOD.B10 control mice are those expressed in the infiltrating leukocytes (See GEO series GSE154739).

We showed that only 12 genes were similarly changed in the pancreata of NOD mice and AA+ individuals (**Figure 6A**). Surprisingly, four of these genes were among the eight disease-relevant genes that we validated to be changed in the pancreata of AA+ individuals. These four genes, *Cadm2*, *Trpm5, Pdk4*, and *Angptl*, are not highly expressed in leukocytes and thus, their expression is not significantly impacted by the presence infiltrating cells in the NOD pancreas. The other three genes validated to be reduced in the pancreata of AA+ individuals, *Clec4d, Rgs16*, and *Fcgr2b*, are abundantly expressed on leukocytes, thus, their expression in the NOD pancreas would be significantly influenced by the high level of insulitis. The *Crh* gene was not present on the mouse microarray.

Our data suggest that increased expression of *CADM2*, and reduced expression of *TRPM5, PDK4*, and/or *ANGPTL4* could play a role in the development of both human T1D and NOD disease by altering mechanisms that control insulin sensitivity, insulin secretion, β-cell and pancreas development, and/or islet cell organization. The *CADM2* gene encodes cell adhesion molecule 2, a mediator of synaptic signaling. GWAS studies have identified SNPs near *CADM2* that are associated with higher expression of CADM2, and the development of obesity (30, 84). In animal models, increased *Cadm2* expression is associated with obesity, while knockout or loss of *Cadm2* prevents obesity, improves insulin sensitivity and protects mice from developing diabetes (31, 84). Interestingly, Cadm2 expression is reduced in obese and insulin-resistant mice by treatment with leptin (84), an adipose hormone that has been suggested as a potential treatment for T1D (85).

The *TRPM5* gene, encodes the transient receptor potential cation channel, subfamily M, member 5, a calcium-activated non-selective channel expressed in β-cells that is an indispensable regulator of insulin secretion (86, 87). In humans, genetic variation within the *TRPM5* locus associates with pre-diabetic phenotypes in subjects who are at risk for developing T2D (38). In mice, loss of *Trpm5* expression results in compromised glucose-stimulated insulin secretion, and maintained elevated blood glucose levels following a glucose challenge (37). TRPM5 has also been shown to mediate insulin secretion by L-arginine and to potentiate glucose-induced insulin secretion by glucagon-like peptide 1 (88, 89).

The *ANGPTL4* gene, encodes angiopoietin-like 4, a protein that is critically involved in maintaining normal glucose and lipid metabolism (49). In the islets, Angptl4 is expressed in glucagon-secreting alpha cells, and enhances glucose-stimulated insulin secretion. Mice deficient in *Angptl4* have impaired glucose tolerance, secreting significantly lower levels of insulin in response to glucose-stimulation, and have dysmorphic islets with abnormally distributed alpha cells (50). Interestingly, we had previously observed disorganization of alpha cell distribution within the islets of 12-week-old NOD mice (14). It is possible that this may result from the significant loss of *Angptl4* expression in the pancreas.

The *PDK4* gene encodes pyruvate dehydrogenase kinase 4. In the pancreas, PDK4 may induce the expression of the pancreas-specific transcription factor 1a (PTF1A) (48). In mice, Ptf1a is involved in pancreatic development and function. Reduced Ptf1a levels leads to pancreatic hypoplasia and glucose intolerance. Ptf1a levels are a determinant of pancreatic size (90). This is interesting, as reduced pancreatic size has been observed in T1D patients, AA+ individuals, and AA- first-degree relatives of T1D patients (7, 8, 91, 92). Ptf1a also activates expression of Pdx1 (Pancreas/duodenum homeobox protein 1) (93, 94), an important transcription factor that regulates β-cell proliferation, differentiation, and function (95). It is possible that reduced levels of PDK4 lead to loss of PTF1A, and subsequently, a loss of PDX1 expression in the pancreata of AA+ individuals and NOD mice.

The *CRH* gene encodes corticotropin-releasing hormone, an important regulator of the hypothalamic-pituitary-adrenal (HPA) axis. In islets, CRH activates corticotropin-releasing hormone receptors on β-cells to stimulate insulin secretion, and promote islet development and proliferation (46, 47, 96). Reduced levels of CRH have been observed in the plasma of T2D patients (97). Reduced pancreatic expression of *CRH* may increase the risk for developing T1D in AA+ individuals.

We observed significantly reduced expression of 3 other genes, *RGS16, CLEC4D*, and *FCGR2B*, in the pancreata of AA+ individuals, but not in that of NOD mice. These genes are enriched in leukocytes, and loss of their expression may contribute to the development of T1D by promoting inflammation, activating T cells and B-cells, and altering β-cell development. The *RGS16* gene encodes regulator of G‐protein signaling 16, and is abundantly expressed in immune cells and pancreatic islets (61, 98–100). Increased RGS16 expression inhibits T cell migration in response to various chemokines, and downregulates pro-inflammatory cytokine production in monocytes (99, 100). In the pancreas, RGS16 is highly enriched in β-cells, where it is involved in the cell development and proliferation (60), and in the regulation of insulin secretion (61).

The *CLEC4D* gene encodes a C-type lectin mycobacterial receptor that is highly expressed in macrophages and myeloid cells. CLEC4D plays a crucial role in anti-mycobacterial host defense. In mice, loss of Clec4d expression results in elevated inflammation, increased mycobacterial levels, and increased mortality (58). In humans, a polymorphism in the *CLEC4D* gene that results in reduced expression, is associated with increased susceptibility to pulmonary tuberculosis (58). T1D patients have a reduced capacity to produce pro-inflammatory cytokines in response to *Mycobacterium tuberculosis* (101), and have a higher risk of developing tuberculosis (102). This may involve a loss of CLEC4D expression.

The *FCGR2B* gene encodes CD32B, an inhibitory low affinity receptor for the Fc region of IgG complexes that is highly expressed on B-cells. This receptor contains an immunoreceptor tyrosine-based inhibitory motif that negatively regulates the BCR complex and its signaling threshold (20). Activation of CD32B in B-cells ultimately leads to diminished BCR-dependent cell activation and antibody production, and loss of CD32B expression can lead to hyper-responsiveness, proliferation and maturation of B-cells. Diminished CD32B expression has already been observed in a number of autoimmune diseases. The autoimmune susceptibility of various strains of mice including NOD mice has also been attributed to polymorphisms in the *FCGR2B* gene that result in reduced CD32B expression in germinal center B-cells (26, 27). Loss of *FCGR2B* gene expression, and reduced surface expression of CD32B have been observed in activated B-cells of NOD mice, and human systemic lupus erythematosus patients (24–27), while a lower frequency of CD32B+ B-cells, and lower mean fluorescent intensity of CD32B expression have been observed in B-cells of rheumatoid arthritis patients compared to controls (23). Reduced *FCGR2B* gene expression has also been observed in the PBMCs of patients with active Graves’ disease (92).

Here, we showed that *FCGR2B* gene expression was significantly reduced in the pancreas and peripheral blood of AA+ progressors and T1D patients compared to controls. *CD19, CD20*, and *CD11c* expression was not significantly changed in AA+ progressors. However, when *FCGR2B* is expressed relative to *CD19* or *CD20* expression, differences between the AA+ progressors vs. AA-FDRs were more significant. This was also observed in T1D patients, suggesting that *FCGR2B* expression may be diminished in B-cells of “pre-diabetic” AA+ individuals and T1D patients. There is abundant evidence suggesting a role for B-cells in T1D (103–106). A loss of CD32B expression may lead to deficient negative feedback regulation of the BCR resulting in intrinsic hyper-responsiveness, proliferation and maturation of B-cells, and contribute to the onset of T1D. Since significantly reduced expression of *FCGR2B* may be seen in AA+ individuals years before the onset of T1D (**Table 2**), this gene could potentially serve as an early biomarker of disease progression, and be used to predict disease onset. Flow cytometry experiments are currently in progress at our lab to study the expression of CD32B in longitudinal blood samples collected from AA+ individuals and healthy controls.

Together, our data show that different pathological events driven by changes in *CADM2*, *TRPM5, PDK4, ANGPTL4, CRH*, *CLEC4D, RGS16*, and *FCGR2B* gene expression may contribute to the development of T1D. These genes are involved in various functions including pancreatic development, β-cell proliferation, insulin sensitivity and secretion, inflammation, and/or immune cell function. Changes in the expression of these genes were mainly observed in the pancreata of AA+ individuals, during the active development of disease, and overlapped well with changes observed in the pancreata of pre-diabetic NOD mice. These findings emphasize the importance of utilizing tissues collected from pre-diabetic individuals, rather than established T1D patients, in understanding the pathogenesis of disease and in identifying early biomarkers of disease progression. Biorepositories such as nPOD and TrialNet play a crucial role in providing access to these rare and important samples.

## Data Availability Statement

The datasets presented in this study can be found in online repositories. The names of the repository/repositories and accession number(s) can be found below: https://www.ncbi.nlm.nih.gov/geo/, GSE72492; https://www.ncbi.nlm.nih.gov/geo/, GSE154739.

## Ethics Statement

The animal study was reviewed and approved by Stanford University Institutional Animal Care and Use Committee (IACUC).

## Author Contributions

LY performed the microarray and QPCR experiments, performed the data analysis, prepared the manuscript, and composed the figures and tables. RF performed RNA extraction, bioanalyzer, and QPCR analysis. RA performed QPCR experiments. CF and LY were involved in the planning and direction of this work. All authors contributed to the article and approved the submitted version.

## Funding

Funding was provided by the National Institute of Health (NIH Grant: 5R01DK11587403) and the Juvenile Diabetes Research Foundation (JDRF grant 2-SRA-2016-340-S-B). This work was also supported by pilot funding from the Stanford Diabetes Research Center (P30DK116074), and a grant from the Myra Reinhard Foundation. This research was performed with the support of the Network for Pancreatic Organ donors with Diabetes (nPOD; RRID:SCR_014641), a collaborative type 1 diabetes research project sponsored by JDRF (nPOD: 5-SRA-2018-557-Q-R) and The Leona M. & Harry B. Helmsley Charitable Trust (Grant#2018PG-T1D053). The content and views expressed are the responsibility of the authors and do not necessarily reflect the official view of nPOD. Organ Procurement Organizations (OPO) partnering with nPOD to provide research resources are listed at http://www.jdrfnpod.org/for-partners/npod-partners/.

## Conflict of Interest

The authors declare that the research was conducted in the absence of any commercial or financial relationships that could be construed as a potential conflict of interest.
