# Supplementary material for: Gene Expression Analysis of the Pre-Diabetic Pancreas to Identify Pathogenic Mechanisms and Biomarkers of Type 1 Diabetes
Source: Front Endocrinol (Lausanne). 2020 Dec 23;11:609271. doi: 10.3389/fendo.2020.609271 (PMC7793767; doi:10.3389/fendo.2020.609271)
Supplement: Supplementary file 1 [file Image_1.pdf]

Supplementary Fig. 1

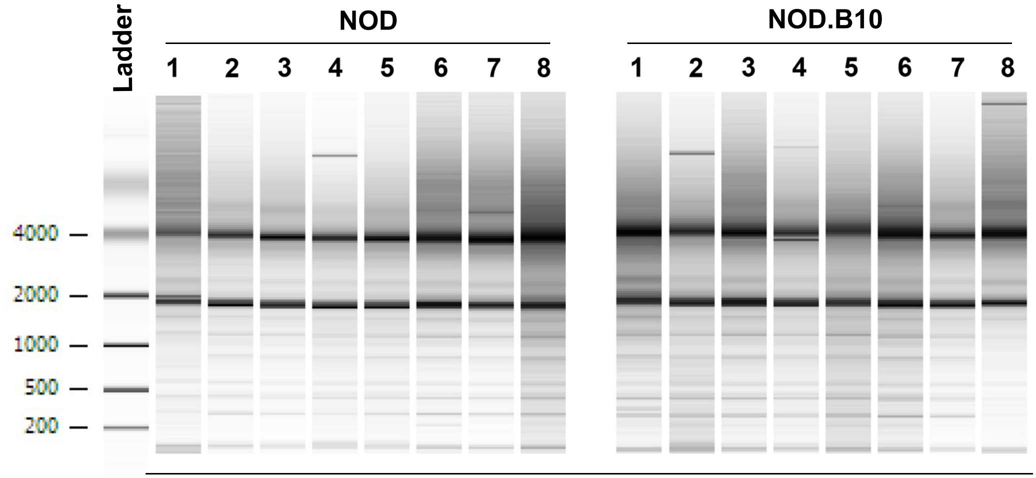

	NOD 1	NOD 2	NOD 3	NOD 4	NOD 5	NOD 6	NOD 7	NOD 8	NOD.B10 1	NOD.B10 2	NOD.B10 3	NOD.B10 4	NOD.B10 5	NOD.B10 6	NOD.B10 7	NOD.B10 8
Blood glucose (mg/dL)	166	122	144	134	155	125	137	143								
Weight (g)	23.5	23.6	23.4	25.6	27.5	23.7	23.6	23.2	26.5	26.0	24.5	25.2	23.7	23.6	25.6	23.5

Supplementary Fig. 1 Bioanalyzer traces of RNA samples extracted from the pancreas of 12 week old NOD and NOD.B10 mice (n=8/group) are shown, along with body weights, and the blood glucose measurements taken from NOD mice prior to sacrifice.
