# Supplementary figures and images for: Gene Expression Analysis of the Pre-Diabetic Pancreas to Identify Pathogenic Mechanisms and Biomarkers of Type 1 Diabetes
Source: Front Endocrinol (Lausanne). 2020 Dec 23;11:609271. doi: 10.3389/fendo.2020.609271 (PMC7793767; doi:10.3389/fendo.2020.609271)

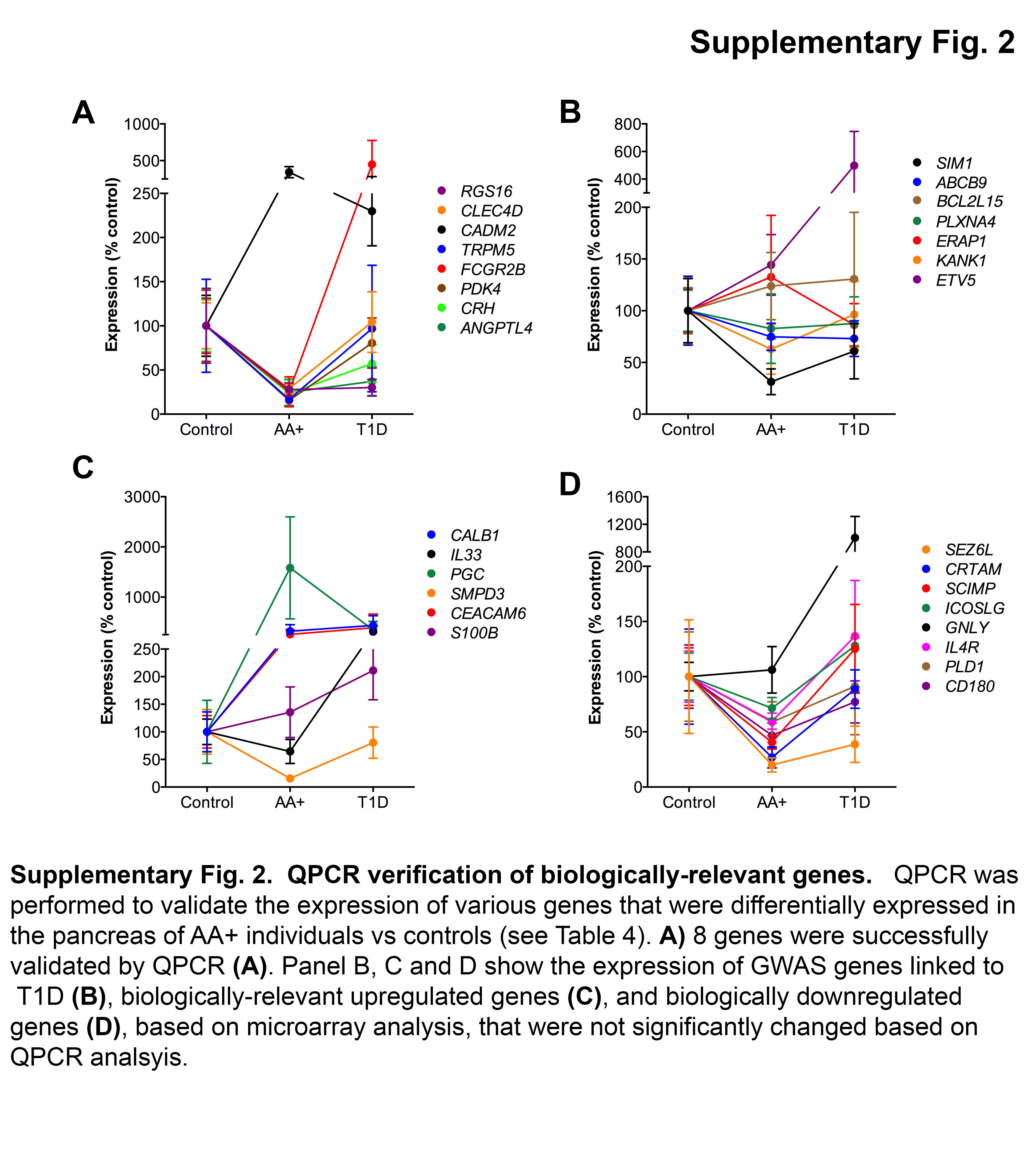

Supplement: Supplementary file 2 [file Image_2.jpeg]
