# Supplementary material for: Gene Expression Analysis of the Pre-Diabetic Pancreas to Identify Pathogenic Mechanisms and Biomarkers of Type 1 Diabetes
Source: Front Endocrinol (Lausanne). 2020 Dec 23;11:609271. doi: 10.3389/fendo.2020.609271 (PMC7793767; doi:10.3389/fendo.2020.609271)
Supplement: Supplementary file 3 [file Image_3.pdf]

Supplementary Fig. 3

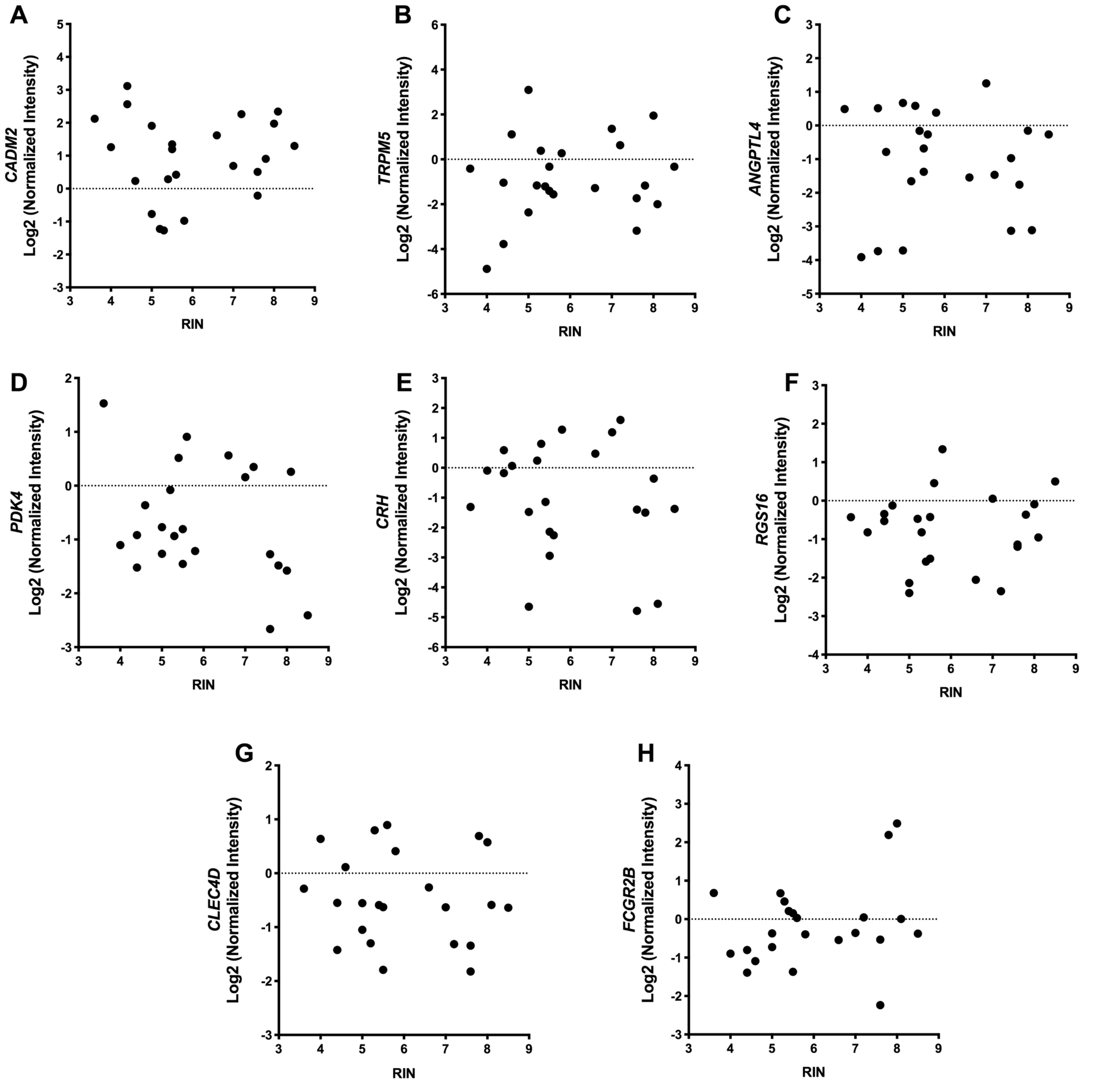

Supplementary Fig. 3. Expression of genes that are differentially expressed in the pancreata of AA+ individuals vs. control, as measured by microarray analysis and shown relative to the RIN value of each sample.
