# Supplementary material for: Gene Expression Analysis of the Pre-Diabetic Pancreas to Identify Pathogenic Mechanisms and Biomarkers of Type 1 Diabetes
Source: Front Endocrinol (Lausanne). 2020 Dec 23;11:609271. doi: 10.3389/fendo.2020.609271 (PMC7793767; doi:10.3389/fendo.2020.609271)
Supplement: Supplementary file 4 [file Image_4.pdf]

Supplementary Fig. 4

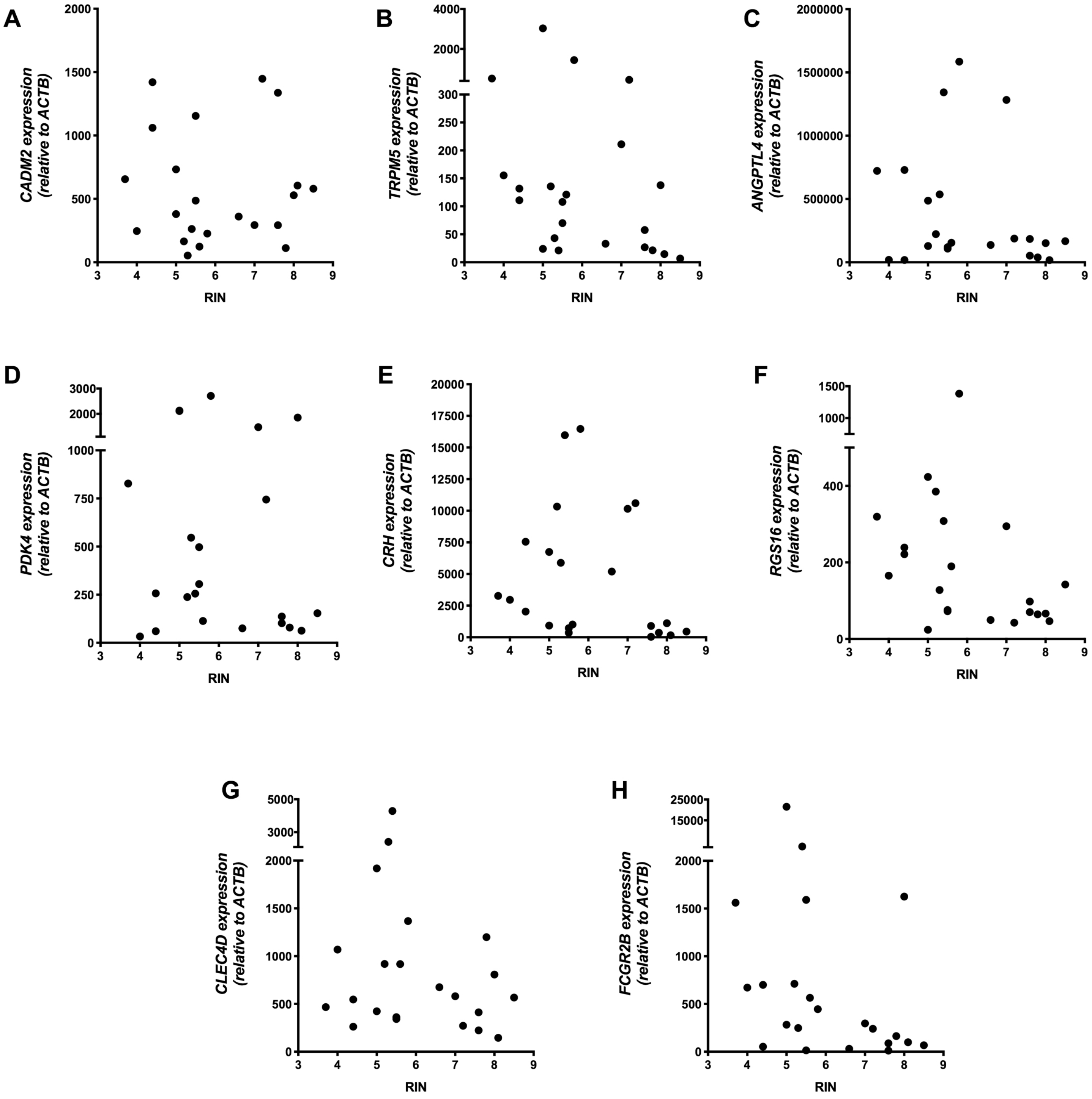

Supplementary Fig. 4. Expression of genes that are differentially expressed in the pancreata of AA+ individuals vs. controls, as measured by QPCR analysis and shown relative to the RIN value of each sample.
