# Supplementary material for: Gene Expression Analysis of the Pre-Diabetic Pancreas to Identify Pathogenic Mechanisms and Biomarkers of Type 1 Diabetes
Source: Front Endocrinol (Lausanne). 2020 Dec 23;11:609271. doi: 10.3389/fendo.2020.609271 (PMC7793767; doi:10.3389/fendo.2020.609271)
Supplement: Supplementary file 5 [file Table_1.docx]

**Supplementary Table 1: Gender-specific genes changed by ≥5-fold in the pancreata of males vs. females**

| **Probe ID** | **Fold change**  **Male vs. Female** | **Gene Symbol** | **Chr.** | **Description** |
| --- | --- | --- | --- | --- |
| A_33_P3341686 | -906.0 | *XIST* | X | X inactive specific transcript |
| A_19_P00329511 | -642.6 | *XIST* | *X* | X inactive specific transcript |
| A_19_P00802872 | -540.6 | *XIST* | X | X inactive specific transcript |
| A_19_P00323692 | -528.0 | *XIST* | X | X inactive specific transcript |
| A_19_P00331623 | -469.8 | *XIST* | X | X inactive specific transcript |
| A_19_P00319151 | -262.8 | *XIST* | *X* | X inactive specific transcript |
| A_19_P00327297 | -179.0 | *XLOC_008015* | *X* | BROAD Institute lincRNA |
| A_19_P00320438 | -15.9 | *TSIX* | X | TSIX transcript, XIST antisense RNA |
| A_19_P00321917 | -9.2 | *TSIX* | *X* | TSIX transcript, XIST antisense RNA |
| A_19_P00326132 | -8.9 | *TSIX* | X | TSIX transcript, XIST antisense RNA |
| A_19_P00316565 | -8.4 | *TSIX* | X | TSIX transcript, XIST antisense RNA |
| A_19_P00317451 | -7.4 | *XIST* | X | X inactive specific transcript |
| A_19_P00326248 | -6.1 | *XLOC_008015* | *X* | BROAD Institute lincRNA |
| A_23_P103588 | -5.4 | *HMGCS2* | *1* | 3-hydroxy-3-methylglutaryl-CoA synthase 2 |
| A_33_P3253598 | 5.0 | *GYG2P1* | *Y* | glycogenin 2 pseudogene 1 |
| A_33_P3284247 | 6.3 | *GYG2P1* | *Y* | glycogenin 2 pseudogene 1 |
| A_33_P3725324 | 9.9 | *USP9Y* | *Y* | ubiquitin specific peptidase 9, Y-linked |
| A_33_P3308232 | 9.9 | *FAM224A* | *Y* | family with sequence similarity 224, A |
| A_24_P130936 | 11.8 | *DDX3Y* | *Y* | DEAD box helicase 3, Y-linked |
| A_33_P3284253 | 13.5 | *GYG2* | *X* | glycogenin 2, transcript variant 4 |
| A_23_P96658 | 14.4 | *TXLNGY* | *Y* | taxilin gamma pseudogene, Y-linked, |
| A_24_P348861 | 14.8 | *TTTY15* | *Y* | testis-specific transcript, Y-linked 15 |
| A_23_P160004 | 16.2 | *UTY* | *Y* | ubiquitously transcribed tetratricopeptide repeat containing, Y-linked |
| A_24_P237511 | 17.3 | *EIF1AY* | *Y* | eukaryotic translation initiation factor 1A, Y-linked |
| A_24_P942743 | 22.4 | *ZFY* | *Y* | zinc finger protein, Y-linked |
| A_33_P3228977 | 27.9 | *UTY* | *Y* | ubiquitously transcribed tetratricopeptide repeat containing, Y-linked |
| A_23_P364792 | 37.0 | *TXLNGY* | *Y* | taxilin gamma pseudogene, Y-linked |
| A_33_P3260223 | 43.1 | *TXLNGY* | *Y* | taxilin gamma pseudogene, Y-linked |
| A_23_P137238 | 52.8 | *KDM5D* | *Y* | lysine specific demethylase 5D |
| A_23_P73848 | 116.5 | *NCRNA00185* | *Y* | non-protein coding RNA 185 |
| A_33_P3224331 | 128.0 | *DDX3Y* | *Y* | DEAD box helicase 3, Y-linked |
| A_33_P3217700 | 174.2 | *USP9Y* | *Y* | ubiquitin specific peptidase 9, Y-linked |
| A_23_P324384 | 1432.1 | *RPS4Y2* | *Y* | ribosomal protein S4, Y-linked 2 |
| A_23_P259314 | 1491.1 | *RPS4Y1* | *Y* | ribosomal protein S4, Y-linked 1 |
