# Supplementary material for: Gene Expression Analysis of the Pre-Diabetic Pancreas to Identify Pathogenic Mechanisms and Biomarkers of Type 1 Diabetes
Source: Front Endocrinol (Lausanne). 2020 Dec 23;11:609271. doi: 10.3389/fendo.2020.609271 (PMC7793767; doi:10.3389/fendo.2020.609271)
Supplement: Supplementary file 6 [file Table_2.docx]

**Supplementary Table 2: Differentially expressed genes in the pancreata of AA+ compared to controls**

| **Upregulated genes in the pancreas of AA+ vs. controls** | | | | |
| --- | --- | --- | --- | --- |
| **Probe ID** | **Gene Symbol** | **Fold Change**  **(vs. control)** | | **Description** |
|  |  | **AA+** | **T1D** |  |
| A_33_P3370714 | *PGC* | 4.8 | 1.8 | progastricsin |
| A_23_P326020 | *CSMD3* | 4.4 | 2.2 | CUB and Sushi multiple domains 3 |
| A_23_P207336 | *PPY* | 4.0 | 1.0 | pancreatic polypeptide |
| A_32_P215943 | *LINC00661* | 3.8 | 1.1 | long intergenic non-protein coding RNA 661 |
| A_23_P68487 | *BMP7* | 3.7 | 2.1 | bone morphogenetic protein 7 |
| A_23_P156445 | *DDX43* | 3.6 | 3.6 | DEAD (Asp-Glu-Ala-Asp) box polypeptide 43 |
| A_23_P139146 | *MS4A8* | 3.4 | 3.5 | membrane-spanning 4-domain, subfamily A,8 |
| A_32_P78816 | *PSPH* | 3.4 | -1.0 | phosphoserine phosphatase |
| A_23_P167159 | *SCRG1* | 3.3 | 5.6 | stimulator of chondrogenesis 1 |
| A_23_P65307 | *SLITRK6* | 3.3 | 3.3 | SLIT and NTRK-like family, member 6 |
| A_19_P00315647 | *LOC100507165* | 3.3 | 3.4 | uncharacterized LOC100507165 |
| A_33_P3353210 | *CADM2* | 3.1 | 2.3 | cell adhesion molecule 2 |
| A_23_P146855 | *MPPED1* | 3.0 | 1.0 | metallophosphoesterase domain containing 1 |
| A_23_P218442 | *CEACAM6* | 2.9 | 1.9 | carcinoembryonic antigen-related cell adhesion molecule 6 |
| A_19_P00329557 | *RPSA* | 2.8 | 2.7 | ribosomal protein SA |
| A_32_P14762 | *OOEP* | 2.8 | 2.9 | oocyte expressed protein |
| A_33_P3408177 | *BCL2L15* | 2.7 | 1.4 | BCL2-like 15 |
| A_33_P3304576 | *KRTAP5-5* | 2.7 | 1.3 | keratin associated protein 5-5 |
| A_23_P43197 | *CALB1* | 2.6 | 1.7 | calbindin 1, 28kDa |
| A_23_P167061 | *PPEF2* | 2.6 | 1.1 | protein phosphatase, EF-hand calcium binding domain 2 |
| A_23_P37127 | *FOXA1* | 2.6 | -1.1 | forkhead box A1 |
| A_32_P126375 | *NHS* | 2.5 | 4.7 | Nance-Horan syndrome |
| A_23_P135381 | *SP5* | 2.5 | 1.9 | Sp5 transcription factor |
| A_23_P143526 | *S100B* | 2.5 | 2.7 | S100 calcium binding protein B |
| A_32_P491499 | *LOC285191* | 2.5 | 1.9 | uncharacterized LOC285191 |
| A_23_P76914 | *SIX1* | 2.5 | 1.7 | SIX homeobox 1 |
| A_33_P3406904 | *LOC101928561* | 2.4 | 1.8 | uncharacterized LOC101928561 |
| A_19_P00322654 | *FENDRR* | 2.4 | 2.1 | FOXF1 adjacent non-coding developmental regulatory RNA |
| A_23_P62642 | *CCDC19* | 2.4 | 1.6 | coiled-coil domain containing 19 |
| A_23_P103720 | *AGMAT* | 2.4 | 1.4 | agmatine ureohydrolase |
| A_23_P10980 | *LPHN3* | 2.3 | 3.0 | latrophilin 3 |
| A_23_P163567 | *SMPD3* | 2.3 | 1.5 | sphingomyelin phosphodiesterase 3, neutral membrane |
| A_23_P366376 | *TDGF1* | 2.3 | 1.8 | teratocarcinoma-derived growth factor 1 |
| A_33_P3745020 | *LOC100126584* | 2.3 | 1.1 | uncharacterized LOC100126584 |
| A_23_P330578 | *LRRC39* | 2.3 | 2.1 | leucine rich repeat containing 39 |
| A_32_P77977 | *UTP11L* | 2.2 | 1.4 | UTP11-like, U3 small nucleolar ribonucleoprotein |
| A_23_P383819 | *TBX3* | 2.2 | 1.2 | T-box 3 |
| A_24_P150931 | *NXPH3* | 2.2 | 2.0 | neurexophilin 3 |
| A_33_P3398406 | *GLP2R* | 2.2 | -1.1 | glucagon-like peptide 2 receptor |
| A_32_P160561 | *DOK6* | 2.2 | 2.2 | docking protein 6 |
| A_23_P27107 | *TM4SF5* | 2.2 | 1.1 | transmembrane 4 L six family member 5 |
| A_33_P3259028 | *TRIM24* | 2.2 | 2.3 | tripartite motif containing 24 |
| A_23_P94434 | *HRCT1* | 2.2 | 2.9 | histidine rich carboxyl terminus 1 |
| A_23_P58676 | *NPR3* | 2.2 | 3.5 | natriuretic peptide receptor 3 |
| A_23_P47614 | *PHLDA2* | 2.2 | -1.3 | pleckstrin homology-like domain, family A, 2 |
| A_23_P107116 | *RNF112* | 2.2 | 1.7 | ring finger protein 112 |
| A_23_P316487 | *H2AFV* | 2.1 | 2.2 | H2A histone family, member V |
| A_33_P3237542 | *KRTAP5-8* | 2.1 | 1.5 | keratin associated protein 5-8 |
| A_33_P3413701 | *ERAP1* | 2.1 | 1.1 | endoplasmic reticulum aminopeptidase 1 |
| A_23_P256948 | *MSC* | 2.1 | 1.8 | musculin |
| A_33_P3227990 | *MBP* | 2.1 | 2.0 | myelin basic protein |
| A_33_P3277659 | *ANKRD30B* | 2.1 | -1.7 | ankyrin repeat domain 30B |
| A_33_P3315698 | *ABHD12B* | 2.1 | 1.2 | abhydrolase domain containing 12B |
| A_23_P91317 | *RAD21L1* | 2.1 | -1.3 | RAD21-like 1 |
| A_32_P183442 | *XLOC_l2_011265* | 2.0 | 1.4 | BROAD Institute lincRNA |
| A_23_P80551 | *KRBOX1* | 2.0 | 1.4 | KRAB box domain containing 1 |
| A_23_P80508 | *PLSCR2* | 2.0 | 1.5 | phospholipid scramblase 2 |
| A_19_P00805954 | *LOC100129461* | 2.0 | 1.4 | uncharacterized LOC100129461 |
| A_33_P3300253 | *PTPN20B* | 2.0 | 1.5 | protein tyrosine phosphatase, non-receptor type 20B |
| A_33_P3256952 | *EGLN3* | 1.9 | 2.3 | egl-9 family hypoxia-inducible factor 3 |
| A_23_P10025 | *NELL2* | 1.9 | 2.3 | NEL-like 2 |
| A_24_P200854 | *HOXA2* | 1.8 | 2.4 | homeobox A2 |
| A_32_P30649 | *ETV5* | 1.8 | 2.4 | ets variant 5 |
| A_33_P3281572 | *CMAHP* | 1.8 | 2.0 | cytidine monophospho-N-acetylneuraminic acid hydroxylase, pseudogene |
| A_23_P3956 | *C1QTNF1* | 1.8 | 2.2 | C1q and tumor necrosis factor related protein 1 |
| A_23_P31945 | *IL33* | 1.7 | 2.9 | interleukin 33 |
| A_32_P25737 | *CHIC1* | 1.7 | 2.4 | cysteine-rich hydrophobic domain 1 |
| A_33_P3240492 | *PHYHIPL* | 1.7 | 2.9 | phytanoyl-CoA 2-hydroxylase interacting protein-like |
| A_19_P00317984 | *SNHG5* | 1.6 | 2.2 | small nucleolar RNA host gene 5,non-coding |

| **Downregulated genes in the pancreas of AA+ vs. controls** | | | | | |
| --- | --- | --- | --- | --- | --- |
| **Probe ID** | **Gene Symbol** | **Fold Change**  **(vs. control)** | | **Description** |  |
|  |  | **AA+** | **T1D** |  |  |
| A_23_P31755 | | *CRH* | -4.2 | -2.2 | corticotropin releasing hormone |
| A_24_P243749 | | *PDK4* | -4.0 | -1.6 | pyruvate dehydrogenase kinase, isozyme 4 |
| A_32_P214178 | | *LINC00319* | -3.8 | -2.7 | long intergenic non-protein coding RNA 319 |
| A_24_P272451 | | *SCIMP* | -3.6 | -2.0 | SLP adaptor and CSK interacting membrane protein |
| A_33_P3295358 | | *ANGPTL4* | -3.6 | -2.6 | angiopoietin-like 4 |
| A_33_P3335177 | | *SFRP4* | -3.2 | -1.5 | secreted frizzled-related protein 4 |
| A_19_P00812062 | | *XLOC_l2_009140* | -3.2 | -3.5 | BROAD Institute lincRNA |
| A_23_P80242 | | *SEZ6L* | -3.0 | -1.9 | seizure related 6 homolog (mouse)-like |
| A_23_P41629 | | *ADAMTS16* | -2.9 | -3.0 | ADAM metallopeptidase with thrombospondin type 1 motif, 16 |
| A_23_P98686 | | *ATHL1* | -2.9 | -1.2 | ATH1, acid trehalase-like 1 |
| A_19_P00321264 | | *UBA6-AS1* | -2.8 | -1.6 | UBA6 antisense RNA 1 |
| A_23_P154708 | | *HAO1* | -2.8 | -2.5 | hydroxyacid oxidase |
| A_32_P228167 | | *C12orf50* | -2.8 | -4.5 | chromosome 12 open reading frame 50 |
| A_33_P3336257 | | *IRX1* | -2.8 | -1.3 | iroquois homeobox 1 |
| A_23_P69699 | | *NPY1R* | -2.8 | -2.1 | neuropeptide Y receptor Y1 |
| A_33_P3369336 | | *GCG* | -2.8 | -1.6 | glucagon |
| A_33_P3330786 | | *LOC728339* | -2.7 | -2.3 | uncharacterized LOC728339 |
| A_23_P104318 | | *DDIT4* | -2.7 | -1.1 | DNA-damage-inducible transcript 4 |
| A_23_P394972 | | *TSPEAR* | -2.6 | -2.3 | thrombospondin-type laminin G domain and EAR repeats |
| A_23_P317667 | | *ICOSLG* | -2.6 | -1.6 | inducible T-cell co-stimulator ligand |
| A_23_P259611 | | *NME8* | -2.6 | -1.9 | NME/NM23 family member 8 |
| A_33_P3422010 | | *DNAH12* | -2.6 | -1.9 | dynein, axonemal, heavy chain 12 |
| A_33_P3389802 | | *LOC101928235* | -2.6 | -2.4 | TFU00175 Transcript Finishing Unit |
| A_19_P00321148 | | *LINC01127* | -2.6 | -2.6 | long intergenic non-protein coding RNA 1127 |
| A_23_P208198 | | *ZNF577* | -2.6 | -1.5 | zinc finger protein 577 transcript variant 3 |
| A_19_P00317706 | | *KANK1* | -2.5 | -1.4 | KN motif and ankyrin repeat domains 1 |
| A_24_P367602 | | *DUSP5P1* | -2.5 | -1.2 | dual specificity phosphatase 5 pseudogene 1 |
| A_23_P429425 | | *ST6GAL2* | -2.5 | -2.3 | ST6 beta-galactosamide alpha-2,6-sialyltranferase 2 |
| A_19_P00318183 | | *LINC01197* | -2.5 | -1.4 | long intergenic non-protein coding RNA 1197 |
| A_33_P3271470 | | *GREB1L* | -2.5 | -1.7 | growth regulation by estrogen in breast cancer-like |
| A_19_P00321578 | | *PLD1* | -2.5 | -1.4 | phospholipase D1, phosphatidylcholine-specific |
| A_24_P564030 | | *BTBD8* | -2.5 | -1.0 | BTB (POZ) domain containing 8 |
| A_24_P68183 | | *EGFEM1P* | -2.5 | -2.5 | EGF-like and EMI domain containing, pseudogene |
| A_33_P3381691 | | *C20orf62* | -2.5 | -1.4 | chromosome 20 open reading frame 62 |
| A_33_P3278755 | | *SULT6B1* | -2.4 | -1.6 | sulfotransferase family, cytosolic, 6B, member 1 |
| A_23_P136504 | | *SLC25A21* | -2.4 | -1.7 | solute carrier family 25, member 21 |
| A_23_P151895 | | *CILP* | -2.4 | -1.5 | cartilage intermediate layer protein, nucleotide pyrophosphohydrolase |
| A_23_P319783 | | *SPAG17* | -2.4 | -2.0 | sperm associated antigen 17 |
| A_23_P84118 | | *CDH18* | -2.4 | -1.9 | cadherin 18, type 2 |
| A_23_P214743 | | *SIM1* | -2.4 | -2.2 | single-minded family bHLH transcription factor 1 |
| A_23_P375922 | | *COL19A1* | -2.4 | -1.4 | collagen, type XIX, alpha 1 |
| A_33_P3397733 | | *FAM154B* | -2.3 | -2.5 | family with sequence similarity 154, member B |
| A_33_P3395651 | | *PCDHGA5* | -2.3 | -1.3 | protocadherin gamma subfamily A, 5 |
| A_33_P3789069 | | *LINC00939* | -2.3 | -1.9 | long intergenic non-protein coding RNA 939, long non-coding |
| A_33_P3227217 | | *SNORA81* | -2.3 | -2.1 | small nucleolar RNA, H/ACA box 81 |
| A_33_P3237379 | | *KCNC1* | -2.3 | -2.1 | potassium voltage-gated channel, Shaw-related subfamily, 1, B |
| A_23_P165239 | | *ZNF208* | -2.2 | -1.8 | zinc finger protein 208 |
| A_33_P3364443 | | *CRNDE* | -2.2 | -2.6 | colorectal neoplasia differentially expressed |
| A_23_P2258 | | *CCDC62* | -2.2 | -1.9 | coiled-coil domain containing 62 |
| A_19_P00322242 | | *GALNT9* | -2.2 | -1.6 | polypeptide N-acetylgalactosaminyltransferase 9 |
| A_23_P87279 | | *TRPM5* | -2.2 | -1.8 | transient receptor potential cation channel, subfamily M, member 5 |
| A_33_P3227904 | | *SLC25A34* | -2.2 | -3.4 | solute carrier family 25, member 34 |
| A_33_P3376947 | | *ABCB9* | -2.2 | -2.1 | ATP-binding cassette, sub-family B, member 9 |
| A_33_P3422787 | | *WWTR1-AS1* | -2.2 | -2.0 | cDNA, FLJ99482 |
| A_23_P209954 | | *GNLY* | -2.2 | 1.8 | granulysin, transcript variant NKG5 |
| A_33_P3724157 | | *DERL3* | -2.2 | -1.9 | derlin 3, transcript variant 3 |
| A_23_P307968 | | *CDH23* | -2.2 | -2.3 | cadherin-related 23 |
| A_19_P00320828 | | *CRYBB2P1* | -2.2 | -1.4 | crystallin, beta B2 pseudogene 1 |
| A_32_P217390 | | *SPATA8* | -2.2 | -2.2 | spermatogenesis associated 8 |
| A_23_P257815 | | *CD180* | -2.2 | 1.1 | CD180 molecule |
| A_33_P3279959 | | *PLXNA4* | -2.2 | 1.2 | plexin A4 |
| A_23_P110712 | | *DUSP1* | -2.2 | -1.2 | dual specificity phosphatase 1 |
| A_32_P105825 | | *MPPED2* | -2.2 | -1.6 | metallophosphoesterase domain containing 2 |
| A_23_P305092 | | *CRTAM* | -2.2 | 1.4 | cytotoxic and regulatory T cell molecule |
| A_23_P24129 | | *DKK1* | -2.1 | -1.5 | dickkopf WNT signaling pathway inhibitor 1 |
| A_33_P3318053 | | *CRHR1-IT1* | -2.1 | -1.4 | CRHR1 intronic transcript 1 non-protein coding |
| A_33_P3587376 | | *SNAR-A3* | -2.1 | -3.9 | small ILF3/NF90-associated RNA A3 |
| A_33_P3210180 | | *TLE1* | -2.1 | -2.2 | clone FP17926 unknown mRNA |
| A_24_P160680 | | *CCDC40* | -2.1 | -2.0 | coiled-coil domain containing 40 |
| A_23_P64306 | | *TRIM48* | -2.1 | -1.9 | tripartite motif containing 48 |
| A_33_P3245606 | | *BHLHA9* | -2.1 | -1.8 | basic helix-loop-helix family, member a9 |
| A_33_P3266730 | | *SYTL4* | -2.1 | -1.3 | synaptotagmin-like 4 |
| A_23_P344281 | | *ZIK1* | -2.1 | -1.7 | zinc finger protein interacting with K protein 1 |
| A_33_P3297562 | | *IRX2* | -2.1 | -1.3 | iroquois homeobox 2, transcript variant 1, |
| A_33_P3258977 | | *CLEC4D* | -2.1 | -1.3 | C-type lectin domain family 4, member D |
| A_23_P419714 | | *BTBD11* | -2.1 | -1.3 | BTB (POZ) domain containing 11 |
| A_23_P34644 | | *FCGR2B* | -2.1 | 1.2 | Fc fragment of IgG, low affinity IIb, receptor |
| A_33_P3329433 | | *TTN* | -2.1 | -2.3 | titin, transcript variant IC |
| A_33_P3289371 | | *PHACTR3* | -2.1 | -1.9 | phosphatase and actin regulator 3 |
| A_33_P3313810 | | *CELA1* | -2.1 | -1.9 | chymotrypsin-like elastase family, member 1 |
| A_32_P217140 | | *ISX* | -2.1 | -3.5 | intestine-specific homeobox |
| A_33_P3378634 | | *SLC25A30-AS1* | -2.1 | -1.7 | SLC25A30 antisense RNA 1 |
| A_19_P00316387 | | *CCDC141* | -2.1 | -2.2 | cDNA clone IMAGE:30376005 |
| A_33_P3295538 | | *AMIGO3* | -2.1 | -2.7 | adhesion molecule with Ig-like domain 3 |
| A_23_P312383 | | *C5orf64* | -2.0 | -1.8 | chromosome 5 open reading frame 64 |
| A_23_P217341 | | *MAGEB1* | -2.0 | -1.7 | melanoma antigen family B, 1 |
| A_32_P21246 | | *HCG27* | -2.0 | -1.1 | HLA complex group 27, non-protein coding |
| A_33_P3610406 | | *ECEL1P2* | -2.0 | -2.0 | endothelin converting enzyme-like 1, pseudogene |
| A_33_P3319593 | | *FBXO31* | -2.0 | -1.5 | F-box protein 31 |
| A_33_P3420900 | | *PATE2* | -2.0 | -1.1 | prostate and testis expressed 2 |
| A_23_P152462 | | *STX1B* | -2.0 | -2.1 | syntaxin 1B |
| A_33_P3395947 | | *IL4R* | -2.0 | -1.9 | interleukin 4 receptor, transcript variant 4 |
| A_23_P338410 | | *WFDC9* | -2.0 | -2.0 | WAP four-disulfide core domain 9 |
| A_33_P3303319 | | *LPPR5* | -2.0 | -1.8 | lipid phosphate phosphatase-related protein type 5 |
| A_24_P101921 | | *EXOC5* | -2.0 | -2.0 | PRO1912 mRNA |
| A_23_P420293 | | *C11orf45* | -2.0 | -1.3 | chromosome 11 open reading frame 45 |
| A_33_P3338674 | | *CCDC163P* | -2.0 | -2.2 | coiled-coil domain containing 163, pseudogene, non-coding RNA |
| A_23_P164068 | | *DNAH9* | -2.0 | -2.1 | dynein, axonemal, heavy chain 9 |
| A_33_P3274627 | | *NOL4* | -1.9 | -2.2 | nucleolar protein 4 |
| A_33_P3212909 | | *ZFHX2* | -1.9 | -2.4 | zinc finger homeobox 2 |
| A_23_P8571 | | *SRCRB4D* | -1.9 | -2.1 | scavenger receptor cysteine rich domain containing, group B |
| A_23_P18751 | | *TMPRSS11E* | -1.8 | -2.0 | transmembrane protease, serine 11E |
| A_33_P3880078 | | *RSU1P2* | -1.8 | -2.6 | Ras suppressor protein 1 pseudogene 2, non-coding |
| A_33_P3400552 | | *DEFB108B* | -1.8 | -2.1 | defensin, beta 108B |
| A_33_P3241696 | | *SLC45A2* | -1.8 | -2.4 | solute carrier family 45, member 2 |
| A_33_P3405479 | | *OR4C45* | -1.8 | -2.3 | olfactory receptor, family 4, subfamily C, 45 |
| A_19_P00315492 | | *LOC101927184* | -1.7 | -2.8 | uncharacterized LOC101927184 |
| A_33_P3555368 | | *FLJ26086* | -1.7 | -2.1 | cDNA FLJ26086 fis |
| A_32_P109683 | | *PAGE2B* | -1.7 | -2.1 | P antigen family, member 2B |
| A_23_P320578 | | *RGS16* | -1.7 | -2.4 | regulator of G-protein signaling 16 |
| A_23_P84736 | | *CTNNA2* | -1.7 | -2.0 | catenin (cadherin-associated protein), alpha 2 |
| A_23_P10172 | | *PRSS50* | -1.7 | -2.0 | protease, serine, 50 |
| A_19_P00316622 | | *LOC102723325* | -1.7 | -2.0 | uncharacterized LOC102723325 |
| A_33_P3386150 | | *NRADDP* | -1.7 | -2.5 | neurotrophin receptor associated death domain, pseudogene |

*QPCR was performed to measure expression of the highlighted genes. See Table 4 for biological significance of these genes.
