# Supplementary material for: Gene Expression Analysis of the Pre-Diabetic Pancreas to Identify Pathogenic Mechanisms and Biomarkers of Type 1 Diabetes
Source: Front Endocrinol (Lausanne). 2020 Dec 23;11:609271. doi: 10.3389/fendo.2020.609271 (PMC7793767; doi:10.3389/fendo.2020.609271)
Supplement: Supplementary file 8 [file Table_4.pdf]

Supplementary Table 4: QPCR cycle threshold values of differentially expressed genes in the pancreata of AA+ vs. controls

cDNA prep 1

	ID	*ACTB*	*ANGPTL4*	*CRH*	*CLEC4D*	*RGS16*	*CD19*	*CD20*	*CD11c*
**Control**	6172	27.79	17.16	23.75	27.34	27.32	32.76	25.34	25.28
	6179	26.75	18.95	23.38	26.87	28.13	31.50	23.73	21.64
	6227	25.58	18.31	25.57	25.71	27.98	32.48	24.95	22.16
	6229	26.70	16.38	23.36	27.49	28.47	33.47	26.95	26.06
	6234	27.64	18.14	25.93	28.73	29.28	n.d.	27.38	27.42
	6253	25.92	16.86	23.37	24.65	28.89	33.66	25.78	22.01
**AA+**	6027	27.39	23.18	26.37	29.32	29.56	n.d.	28.72	26.30
	6090	25.23	18.48	26.71	26.78	28.96	30.83	24.03	23.18
	6123	26.46	20.75	26.62	27.74	29.82	33.46	26.79	23.72
	6170	26.24	18.71	31.02	28.40	30.07	32.56	25.91	24.61
	6184	27.63	18.12	24.72	28.51	29.70	33.91	26.32	24.39
	6197	24.74	20.69	27.41	27.52	29.16	30.14	22.54	24.28
**T1D**	6088	25.97	19.08	26.46	27.44	29.76	22.14	15.68	24.86
	6143	24.39	17.01	25.56	25.21	27.21	31.31	22.02	22.81
	6148	29.97	22.97	27.21	29.03	31.21	n.d.	27.42	27.07
	6161	25.54	16.61	25.66	26.78	30.92	32.79	26.06	26.08
	6180	27.49	23.21	25.92	27.39	30.08	33.64	24.44	23.30
	6241	24.50	17.26	24.35	24.81	28.41	20.60	15.09	22.65
	6245	26.20	18.65	22.79	28.08	30.76	30.65	23.47	25.35
	6258	27.99	17.60	23.99	25.89	29.69	29.56	23.84	25.60
	6263	24.00	18.70	25.52	23.74	27.95	28.95	21.45	19.88
	6266	26.21	19.11	23.83	26.78	30.57	31.76	24.33	24.72

cDNA prep 2

	ID	*ACTB*	*TRPM5*	*PDK4*	*FCGR2B*
**Control**	6172	25.99	25.46	24.55	27.15
	6179	23.97	26.85	26.04	24.46
	6227	23.40	26.45	26.53	24.22
	6229	24.06	26.30	23.50	25.80
	6234	26.03	26.97	26.30	25.38
	6253	23.64	28.17	24.51	25.65
**AA+**	6027	25.56	28.73	29.61	29.80
	6090	23.95	27.78	25.66	30.05
	6123	25.12	29.24	27.99	28.64
	6170	22.97	28.19	26.27	29.34
	6184	25.01	27.93	26.97	25.52
	6197	22.17	28.27	26.16	25.50
**T1D**	6088	25.51	28.72	26.52	24.84
	6143	22.03	29.27	24.73	25.93
	6148	29.69	28.09	28.61	25.26
	6161	22.06	27.45	20.97	23.89
	6180	25.71	28.40	30.63	26.29
	6241	23.17	26.03	22.28	22.47
	6245	23.95	25.08	24.37	26.00
	6258	23.91	29.48	25.88	22.62
	6263	21.79	27.34	25.45	24.40
	6266	22.07	26.98	25.79	27.07

cDNA prep 3

	ID	*ACTB*	*18S*	*GAPDH*	*CADM2*
**Control**	6172	27.07	13.88	27.98	29.22
	6179	26.31	13.53	25.84	28.91
	6227	25.81	14.81	26.34	28.83
	6229	26.37	13.87	26.63	28.16
	6234	28.73	15.43	28.23	29.34
	6253	26.01	14.16	26.02	30.26
**AA+**	6027	27.85	14.54	27.67	27.76
	6090	26.29	14.55	26.90	27.33
	6123	27.57	15.52	26.72	27.15
	6170	25.44	13.46	27.17	27.21
	6184	27.44	14.72	27.93	26.94
	6197	24.31	13.20	24.63	25.04
**T1D**	6088	27.09	14.51	27.38	26.89
	6143	24.72	13.32	24.45	25.51
	6148	29.84	14.95	30.43	30.29
	6161	24.61	13.16	25.24	26.01
	6180	27.13	14.88	28.09	29.15
	6241	25.21	14.26	26.05	26.13
	6245	26.31	13.85	25.86	25.78
	6258	26.55	13.61	25.41	28.48
	6263	23.96	13.59	24.86	27.11
	6266	24.69	11.88	24.21	26.16
